# Clinical Outcome Associated With Beta‐Lactam Allergy Labels in Hospitalized Patients in Belgium

**DOI:** 10.1002/clt2.70166

**Published:** 2026-03-27

**Authors:** Liesbeth Gilissen, Greet Van De Sijpe, Annouschka Laenen, Peter Declercq, Dries Wets, Ileana Ghiordanescu, Anca Mirela Chiriac, Willy E. Peetermans, Paul De Munter, Isabel Spriet, Rik Schrijvers

**Affiliations:** ^1^ Department of Microbiology, Immunology and Transplantation, Allergy and Clinical Immunology Research Group KU Leuven Leuven Belgium; ^2^ Department of Pharmaceutical and Pharmacological Sciences, Clinical Pharmacology and Pharmacotherapy KU Leuven Leuven Belgium; ^3^ Pharmacy Department University Hospitals Leuven Leuven Belgium; ^4^ KU Leuven Biostatistics and Statistical Bioinformatics Centre (L‐BioStat) Leuven Belgium; ^5^ Department of General Internal Medicine, Allergy and Clinical Immunology Unit University Hospitals Leuven Leuven Belgium; ^6^ Laboratoire Inserm UMR1327 ISCHEMIA Tours France; ^7^ Transversal Allergy Unit University Hospital of Tours (CHRU de Tours) Tours France; ^8^ Division of Allergy, Department of Pulmonology Hôpital Arnaud de Villeneuve, University Hospital of Montpellier Montpellier France; ^9^ IDESP, UMR UA11, University of Montpellier—INSERM Montpellier France; ^10^ Department of Microbiology, Immunology and Transplantation, Laboratory for Clinical Infectious and Inflammatory Disorders KU Leuven Leuven Belgium; ^11^ Department of General Internal Medicine, Infectiology Unit University Hospitals Leuven Leuven Belgium

**Keywords:** antimicrobial stewardship, beta‐lactams, delabeling, drug allergy, penicillin allergy

## Abstract

**Background:**

In the United States, beta‐lactam allergy labels (BLAL) are documented in 9%–16% of hospitalized patients and associated with worse clinical outcomes such as increased mortality, length of hospital stay (LOS), intensive care unit (ICU) admission, and use of alternative antibiotics, providing an incentive for broad delabeling protocols. In Europe, BLAL prevalences are lower (0.6%–5%) and the association with clinical outcomes insufficiently explored. Therefore, we assessed the association between BLAL and penicillin allergy label (PenAL) and clinical outcomes and antibiotic use in hospitalized patients in Belgium.

**Methods:**

Retrospective population‐based cohort study of all patients admitted to the University Hospitals Leuven between 2010 and 2018 for pneumonia, pyelonephritis (therapeutic indications), or appendectomy, coronary artery bypass grafting, total knee or hip replacement (prophylactic indications) or heart, kidney, liver, or lung transplantation (mixed indications). Multivariable regression analysis was performed, using BLAL or PenAL as independent variable, and age, gender, Charlson Comorbidity Index, and diagnosis as a priori hypothesized confounders.

**Results:**

We included 21,999 patients accounting for 23,842 admissions. A BLAL was recorded in 1394 (6.3%) patients, with 1113 (5.1%) having an unspecified PenAL. An increased use of next‐line antibiotics was observed among patients with BLAL or PenAL. However, BLAL or PenAL were not associated with altered in‐hospital or 3‐month post‐hospitalization mortality, LOS or ICU admission.

**Conclusion:**

Despite altered antibiotic use, we observed no association of BLAL or PenAL with clinical outcome parameters, highlighting regional differences and limiting transferability of non‐EU findings to guide EU delabeling protocols.

**Abbreviations**
AL: Allergy label
BEH‐SAC: Belgian Hospitals surveillance of antimicrobial consumption
BL: Beta‐lactams
BLAL: Beta‐lactam allergy label
C. difficile: *Clostridium difficile*
CI: Confidence interval
EAACI: European Academy of Allergy and Clinical Immunology
EPR: Electronic patients records
GEE: Generalized estimating equations
HCT: Hematopoietic cell transplant
HR: Hazard ratio
ICD: International classification of disease
ICU: Intensive care unit
IRR: Incidence rate ratio
LOS: Length of hospital stay
MRSA: Methicillin‐resistant *Staphylococcus aureus*
OR: Odds ratio
SOT: Solid organ transplant
UK: United Kingdom
UZ Leuven: University Hospitals Leuven
VRE: Vancomycin‐resistant *Enterococcus*

## 1 Introduction

Beta‐lactam (BL) allergies are among the most frequently reported adverse drug reactions [1]. In US studies, the prevalence of BL allergy labels (BLAL) reported in electronic patient records (EPR) ranges between 9% and 16% [2, 3, 4, 5, 6], whereas European studies report a significantly lower prevalence of 0.6%–5% [7, 8, 9, 10]. In our center, covering over 1 million patients, the previously reported BLAL prevalence was 2%, with rates varying from 1.0% in outpatient settings to 6.0% among hospitalized patients [11].

Allergy labels by themselves can influence therapeutic choices, which in turn may influence clinical outcomes. Most studies conducted outside of Europe indicate that BLAL have a negative impact on patient care [12]. These link BLAL with worse clinical outcomes, such as increased length of hospital stay (LOS) and increased intensive care unit (ICU) admission [5, 13, 14]. Moreover, a higher risk for hospital readmission within 6 weeks was reported in Australia [15]. BLAL were also associated with higher inpatient [5, 14] and outpatient [16] mortality rates. Penicillin allergy labels (PenAL), in particular, were associated with increased death within 30 and 180 days post‐hospitalization [14] and increased odds of infection with antibiotic‐resistant micro‐organisms, such as *Clostridium difficile (C. difficile)* [17], vancomycin‐resistant *Enterococcus* (VRE) [13], and methicillin‐resistant *Staphylococcus aureus* (MRSA) [18]. Additionally, BLAL have led to more use of alternative antibiotics, such as vancomycin, clindamycin, aminoglycosides and quinolones, which can be less effective and/or more toxic [19]. Some studies report higher number of antibiotics used [14, 20], longer antibiotic duration [14] and delays in time‐to‐first‐antibiotic dose [21]. However, there are two US studies reporting no significant differences in antimicrobial treatment duration, hospital length of stay [22] or readmission rates [23] in children with BLAL versus those without. Additionally, a study on liver transplant recipients found no significant differences in mortality, LOS, or hospital readmission rates between those with BLAL and those without [24].

European studies have primarily focused on PenAL [8, 9, 10, 25, 26]. In Portugal, a case‐control study found that patients with PenAL have longer LOS and more frequent infections with MRSA, VRE and *Escherichia coli*, though their mortality rates were lower [25]. In Spain, a nationwide cohort study likewise reported that hospitalized adults with a PenAL had longer hospital stays but did not show higher in‐hospital mortality compared with those without a label [26]. In the United Kingdom (UK), PenAL were associated with increased prescription of non‐penicillin antibiotics and 5.5% increase in LOS [27]. In the Netherlands, patients with PenAL were more likely to be readmitted within 12 weeks and receive next‐line antibiotics [8]. Both European and non‐European studies have highlighted the increased healthcare costs associated with BLAL [7, 28, 29, 30].

The associated risks and costs have fueled the introduction of hospital‐wide delabeling initiatives in the United States and Australia aiming to improve PenAL documentation [31, 32, 33], while European initiatives have only followed afterward in recent years [34, 35, 36]. However, the clinical impact of BLAL and PenAL in Europe, where its prevalence is lower, seems more heterogenous. Moreover, there is uncertainty whether these initiatives can effectively reverse the negative outcomes associated with PenAL. The aim of this study was to assess the association between BLAL and PenAL and clinical outcomes and antibiotic use in the largest Belgian tertiary center. We used a population‐based cohort design including all patients admitted for specific disorders that required therapeutic and/or prophylactic antibiotic treatment, while correcting for potential confounders (age, gender, and Charlson Comorbidity Index), and comparing patients without and with BLAL or PenAL.

## 2 Methods

### 2.1 Study Design, Setting and Patients

We performed a retrospective, population‐based cohort study including all patients hospitalized at the University Hospitals Leuven (UZ Leuven) between February 1, 2010 and December 31, 2018. This period corresponds to the implementation of the current allergy registration and notification module in the EPR, as detailed in the Supporting Information S1. The study included patients hospitalized for at least one of the following conditions: pneumonia, pyelonephritis, appendectomy, coronary artery bypass grafting, total knee or hip replacement, and heart, kidney, liver and/or lung transplantation, who received antibiotics during their hospital stay. Common infections and interventions were chosen to reduce heterogeneity and improve the potential to extrapolate our findings to non‐academic hospitals.

Eligible patients were identified through a retrospective search of the relevant International Classification of Disease (ICD) codes (ICD9 or, from January 2015 onwards, ICD10, see Supporting Information S1: Table E1) among the admission diagnoses registered in the patient's EPR. These codes, available at patient discharge, were combined with antibiotic usage data retrieved from the pharmacy data warehouse. The index event was defined as the date of hospital admission.

The primary outcomes included clinical metrics, that is in‐hospital mortality, 3‐month post‐hospitalization mortality, LOS and ICU admission, and were queried from the EPR. With the exception of LOS, which was calculated as date of discharge minus date of admission plus one, the primary outcomes were categorized as binary outcomes. Secondary outcomes focused on antibiotic use, such as the choice of antibiotics and their class categorized as binary outcomes, the number of different antibiotics administered per admission and the duration of the antibiotic treatment.

The study was approved by the Ethical committee at UZ/KU Leuven.

Data analysis was conducted using SPSS (IBM SPSS Statistics 20, Chicago, IL) and SAS software (version 9.4 of the SAS System for Windows).

### 2.2 Clinical Outcomes

We conducted multivariable analysis to assess the primary outcome variables. The independent variable was the presence of a BLAL in the allergy registration and notification module prior to or during the admission. This was defined as a general allergy label to penicillin, cephalosporin, carbapenem, monobactam, or overall BL antibiotics, or a specific allergy label to a particular antibiotic belonging to the BL class. For details, please see Supporting Information S1: Table E2. The BLAL in our allergy registration were mostly unconfirmed [11]. Age, gender, and Charlson Comorbidity Index (CCI, Sundararajan et al. adaption 2004, [37]) were a priori hypothesized as potential confounders. A two‐tailed *p*‐value of < 0.05 was considered statistically significant. When patients with different diagnoses were grouped, the diagnosis was also added to the model as confounder. Dummy variables were used to enable patients displaying multiple diagnoses. We used logistic regression and computed odds ratio (OR) with associated 95% confidence interval (CI) to analyze mortality and ICU admission rate. To account for data clustering in patients with repeated hospitalizations, generalized estimating equations (GEE) were applied, when more than 3% of observations were correlated, using a robust estimator and an exchangeable working correlation matrix. For LOS, Cox proportional hazard models were applied, accounting for censoring due to in‐hospital death and, when needed, for clustering, using the Lin and Wei method [38]. Additionally, we computed hazard ratios (HR) with 95% CI for the relationship between BLAL and time to hospital discharge.

Subgroup analyses were performed for the following categories: (i) patients receiving antibiotics for therapeutic purposes (i.e., pyelonephritis, pneumonia, transplantation); (ii) patients receiving antibiotics for prophylactic purposes (i.e., appendectomy, coronary artery bypass grafting, prosthesis); (iii) transplant recipients; (iv) ICU‐admitted patients (indicating generally more severe conditions); (v) each individual condition; and (vi) patients with PenAL. Exploratory subanalyses were performed stratified by antibiotic exposure, categorized as BL only, non–BL only, and both BL and non–BL during the same admission.

To reduce the impact of recurrent admissions on in‐hospital and 3‐month post‐hospitalization mortality, a sensitivity logistic regression analysis included only the first hospitalization per patient. A second sensitivity analysis considered only patients with an allergy label documented prior to admission.

### 2.3 Antibiotic Use

The choice of the antibiotic and antibiotic class was assessed using multivariable logistic regression analysis as described earlier. OR were calculated and reported along with the corresponding 95% CI.

The number of different antibiotics used per admission and treatment duration were analyzed using a multivariable Poisson model, with LOS included as an offset variable. The incidence rate ratio (IRR) was reported along with the associated 95% CI. Multiple prescriptions of the same antibiotic were counted only once per admission.

## 3 Results

### 3.1 Study Population

A total of 21,999 patients were included in the study, being 12,585 (57%) men and 9414 (43%) women (Table 1). Among these, 1394 (6.3%) had a BLAL recorded in their EPR. Specifically, 5.5% had a PenAL, with 5.0% having an unspecified general PenAL, and 0.5% had a cephalosporins AL, with 0.4% having an unspecified general cephalosporins AL. Carbapenem and monobactams AL were rare, reported in 0.03% and 0.01% of cases, respectively. For details concerning the prevalence of AL, please consult Supporting Information S1: Table E2. Of the patients with a BLAL, 555 (40%) were men and 839 (60%) were women. A significantly higher proportion of women was observed in this group as compared to that in the no‐BLAL group (60% vs. 42%; *p* < 0.001). Moreover, patients with BLAL were older (mean age 64.6 (± 17.8), versus. 60.7 (± 23.6) in no‐BLAL group), and had greater prevalence of comorbidities (mean CCI 3.58 (± 3.51), vs. 3.33 (± 3.41) in no‐BLAL group). A total of 7.2% of the subjects were hospitalized repeatedly during the study period, resulting in 23,842 admissions (please see Supporting Information S1: Table E3).

**TABLE 1:** Characteristics of the study population.

	No BLAL	BLAL	Total	*p* value
	*N* = 20,605 (93.7)	*N* = 1394 (6.30)	*N* = 21,999 (100)	
Patient characteristics (at first admission)				
Male sex (%)	12,030 (58.4)	8575 (39.8)	12,585 (57.2)	**< 0.001**
Mean age (SD)	60.7 (± 23.6)	64.6 (± 17.8)	61.0 (± 23.3)	**< 0.001**
Mean CCI score (SD)	3.33 (± 3.41)	3.58 (± 3.51)	3.34 (± 3.42)	**< 0.001**
Renal disease, +2 (%)	5814 (28.22)	365 (26.18)	6179 (28.09)	0.506
Any malignancy, including lymphoma and leukemia, except malignant neoplasm of skin, +2 (%)	5680 (27.57)	356 (25.54)	6036 (27.44)	0.530
Chronic pulmonary disease, +1 (%)	5577 (27.07)	358 (25.68)	5935 (26.98)	0.298
Congestive heart failure, +1 (%)	5355 (25.99)	314 (22.53)	5669 (25.77)	0.666
Cerebrovascular disease, +1 (%)	3959 (19.21)	216 (15.49)	4175 (18.98)	0.177
Mycocardial infarction, +1 (%)	3455 (16.77)	172 (12.34)	3627 (16.49)	**0.019**
Peripheral vascular disease, +1 (%)	3000 (14.56)	188 (13.49)	3188 (14.49)	0.652
Metastatic solid tumor, +6 (%)	2558 (12.41)	165 (11.84)	2723 (12.38)	0.443
Mild liver disease, +1 (%)	2362 (11.46)	142 (10.19)	2504 (11.38)	1.000
Diabetes without chronic complication, +1 (%)	1725 (8.37)	108 (7.75)	1833 (8.33)	0.752
Peptic ulcer disease, +1 (%)	1242 (6.03)	78 (5.6)	1320 (6)	0.780
Hemiplegia or paraplegia, +2 (%)	1200 (5.82)	64 (4.59)	1264 (5.75)	0.375
Dementia, +1 (%)	1083 (5.26)	52 (3.73)	1135 (5.16)	0.126
Rheumatic disease, +1 (%)	805 (3.91)	66 (4.73)	871 (3.96)	**0.021**
Moderate to severe liver disease, +3 (%)	763 (3.7)	44 (3.16)	807 (3.67)	0.832
AIDS/HIV, +6 (%)	46 (0.22)	4 (0.29)	50 (0.23)	0.69
Diabetes with chronic complication, +2 (%)	1 (0.005)	0 (0)	1 (0.005)	1.000

	**No BLAL**	**BLAL**	**Total**	*p* value
	***N* = 22,290 (93.49)**	***N* = 1552 (6.51)**	***N* = 23,842 (100)**	
Diagnosis (all admissions)				**< 0.001**
Therapeutic indications (%)	15,588 (69.93)	1053 (67.85)	16,641 (69.79)	0.152
Pneumonia (%)	13,536 (60.73)	897 (57.80)	14,433 (60.54)	**0.033**
Pyelonephritis (%)	1147 (5.15)	81 (5.22)	1228 (5.15)	0.763
Transplantation (%)	1028 (4.61)	93 (5.99)	1121 (4.70)	**0.010**
Heart transplantation (%)	106 (0.48)	6 (0.39)	112 (0.47)	0.604
Lung transplantation (%)	251 (1.13)	43 (2.77)	294 (1.23)	**< 0.001**
Liver transplantation (%)	320 (1.44)	18 (1.16)	338 (1.42)	0.496
Kidney transplantation (%)	386 (1.73)	30 (1.93)	416 (1.74)	0.567
Prophylactic indications (%)	6912 (31.01)	511 (32.93)	7423 (31.13)	0.152
Appendectomy (%)	1789 (8.03)	107 (6.89)	1896 (7.95)	0.105
Coronary artery bypass grafting (%)	2288 (10.26)	146 (9.41)	2434 (10.20)	0.305
Prosthesis (%)	2835 (12.72)	258 (16.62)	3093 (12.97)	**< 0.001**
Hip prosthesis (%)	1278 (5.73)	117 (7.54)	1395 (5.85)	**0.002**
Knee prosthesis (%)	1557 (6.99)	141 (9.09)	1696 (7.12)	**0.002**
ICU admissions (%)	6901 (30.96)	450 (28.99)	7351 (30.83)	0.179
Outcomes (all admissions)				
Mortality (%)	1969 (8.8)	119 (7.7)	2088 (8.8)	0.117
3 months mortality (%)	1267 (5.7)	89 (5.7)	1356 (5.7)	0.931
Length of stay (± SD)	19.92 (± 31.62)	21.57 (± 39.40)	20.03 (± 23,842)	**0.051**
ICU admission (%)	6901 (31.0)	450 (29.0)	7351 (30.8)	0.105

*Note:* values are expressed as % unless otherwise specified. *p*‐values < 0.05 are displayed in bold.

Abbreviations: BLAL, beta‐lactam allergy label; CCI, Charlson comorbidity index; ICU, intensive care unit; SD, standard deviation.

### 3.2 Clinical Outcomes

**FIGURE 1:**
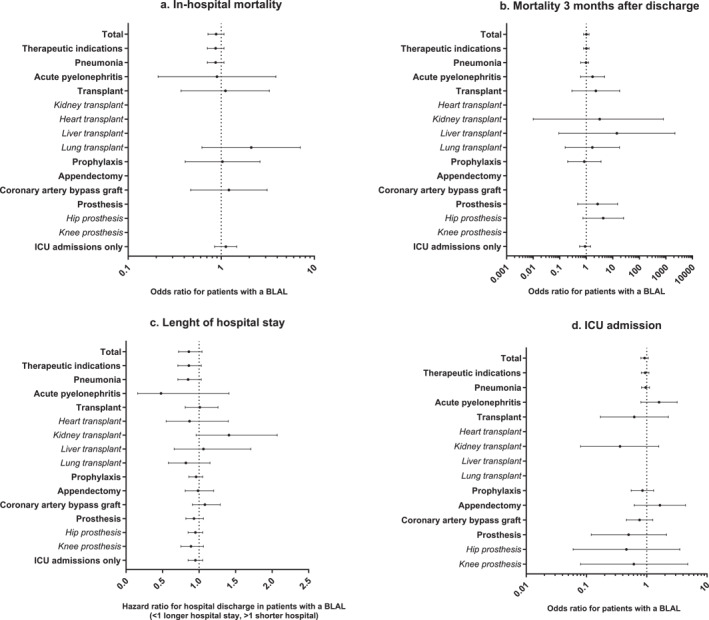
Clinical outcomes associated with a beta‐lactam allergy label (BLAL). Odds ratios (OR) calculated by multivariable regression analysis, with BLAL allergy as independent variable, and age, gender, Charlson Comorbidity Index (CCI) and diagnosis as a priori hypothesized confounders, and use of generalized estimating equations (GEE) to account for data clustering when appropriate; Hazard ratio (HR) calculated by a Cox proportional hazard mode, with length of hospital stay calculated as time between hospital admission and hospital discharge (HR < 1 indicates a lower chance of discharge, hence a longer length of hospital stay).

In the univariate analysis presented in Table 1, the association between BLAL and longer LOS was found to be borderline significant (*p* = 0.051). However, BLAL was also associated with higher disease burden. After adjusting for confounding variables, we observed no significant association between BLAL and LOS. Additionally, BLAL did not show a significant association with increased in‐hospital mortality, 3‐month post‐hospitalization mortality, or ICU admissions, including within diagnostic subgroups, as illustrated in Figure 1 and detailed in the Supporting Information S1: Table E4a. Sensitivity analyses restricted to the first hospitalization only and to patients with BLAL documented prior to hospitalization yielded results consistent with the primary analyses (Supporting Information S1: Table E4b and c). Subanalysis of patients with PenAL showed similar outcomes and is presented in the Supporting Information S1: Table E5.

### 3.3 Antibiotic Use

**FIGURE 2:**
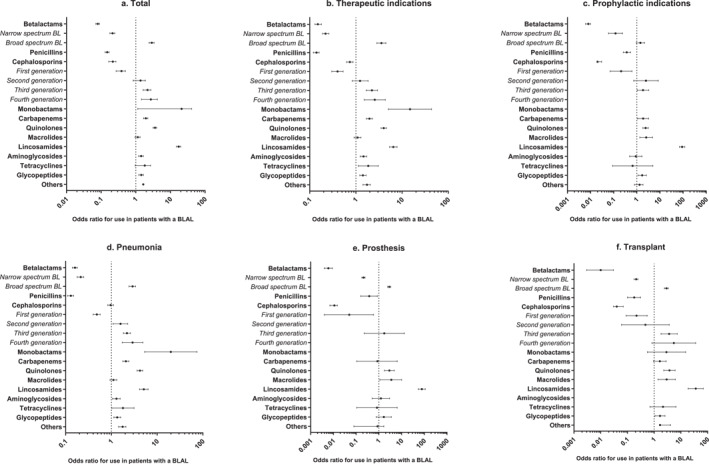
Association of reported beta‐lactam allergy label (BLAL) with choice of antibiotic (class) in the different subpopulations. Odds ratios (OR) calculated by multivariable regression analysis, with BLAL allergy as independent variable, and age, gender, Charlson Comorbidity Index (CCI) and diagnosis as a priori hypothesized confounders, and use of generalized estimating equations (GEE) to account for data clustering when appropriate. Narrow spectrum: benyzlpenicillin, phenoxymethylpenicillin, benzathine benzyl penicillin, amoxicillin, ampicillin, flucloxacillin, amoxicillin/clavulanic acid, cefazolin, cefadroxil, cefuroxime; Broad spectrum: temocillin, piperacillin/tazobactam, cefoxitin, cefotaxime, ceftazidime with/without avibactam, ceftriaxone, cefepime, aztreonam, meropenem, imipenem/cilastatin.

BLAL were associated with a significant reduction in the use of BL antibiotics (59% vs. 95%; OR 0.08, 95% CI [0.07–0.09], *p* < 0.001), a decline which was mainly attributed to a decreased use of penicillins (25% vs. 59%; OR 0.15, 95% CI [0.13–0.17], *p* < 0.001) and cephalosporins (35% vs. 55%; OR 0.21, 95% CI [0.17–0.27], *p* < 0.001). For details, please see Figure 2a, and Supporting Information S1: Table E6. Notably, while first‐generation cephalosporin use decreased, there was an increase in the use of third and fourth‐generation cephalosporins. Additionally, patients with a BLAL exhibited higher usage of monobactams (aztreonam; 0.6% vs. 0.04%; OR 14.82, 95% CI [5.05–43.47], *p* < 0.001) and, to a lesser extent, carbapenems (meropenem; 20% vs. 13%; OR 1.94, 95% CI [1.68–2.24], *p* < 0.001). Furthermore, there was an increase in the use of non‐BL antibiotics, including clindamycin, quinolones, tetracyclines, vancomycin, and aminoglycosides, as detailed in the top 10 antibiotics comparison (Table 2).

**TABLE 2:** Top 10 most frequently used antibiotics in patients without a beta‐lactam allergy label versus with a beta‐lactam allergy label.

No BLAL (*n* = 22,290)	%	BLAL (*n* = 1171)	%
a. Total study population			
Cefazolin	40.6	**Clindamycin**	32.3
Amoxicillin/clavulanic acid	36.1	**Moxifloxacin**	28.2
Piperacillin/tazobactam	25.7	**Levofloxacin**	20.9
**Levofloxacin**	13.1	Meropenem	20.5
Meropenem	12.8	Cefazolin	19.6
**Vancomycin**	11.4	Piperacillin/tazobactam	15.9
Amoxicillin	11.2	**Vancomycin**	14.6
Ceftriaxone	8.6	**Amikacin**	11.6
**Sulfamethoxazole/trimethoprim**	8.3	Ceftriaxone	11.1
**Amikacin**	8.3	**Sulfamethoxazole/trimethoprim**	10.8

**No BLAL (*n* = 15,588)**	%	**BLAL (*n* = 1053)**	%
b. Therapeutic indications			
Amoxicillin/clavulanic acid	47.4	**Moxifloxacin**	40.8
Piperacillin/tazobactam	34.0	Meropenem	29.1
Meropenem	17.6	**Levofloxacin**	24.6
Cefazolin	17.2	Piperacillin/tazobactam	21.9
**Levofloxacin**	15.9	**Vancomycin**	19.3
Amoxicillin	15.5	**Amikacin**	16.3
**Vancomycin**	15.0	**Clindamycin**	16.1
Ceftriaxone	12.1	Ceftriaxone	16.0
**Sulfamethoxazole/trimethoprim**	11.6	**Sulfamethoxazole/trimethoprim**	15.5
**Moxifloxacin**	11.5	Amoxicillin/clavulanic acid	14.1

**No BLAL (*n* = 6912)**	%	**BLAL (*n* = 511)**	%
c. Prophylactic indications			
Cefazolin	93.9	**Clindamycin**	65.4
**Metronidazole**	19.8	Cefazolin	41.3
Amoxicillin/clavulanic acid	10.5	**Metronidazole**	16.4
Piperacillin/tazobactam	7.34	**Levofloxacin**	13.5
**Levofloxacin**	6.90	**Vancomycin**	5.58
**Vancomycin**	3.40	Piperacillin/tazobactam	4.11
**Clindamycin**	2.36	Meropenem	3.33
**Meropenem**	1.87	**Ornidazole**	3.33
Ceftazidime	1.80	Amoxicillin/clavulanic acid	2.55
Flucloxacillin	1.32	**Moxifloxacin**	1.96

*Note:* Multiple prescriptions of the same antibiotic were counted only once per admission; non‐beta‐lactam antibiotics are displayed in bold, the top 10 in Table 2a represents 73% and 71% of total antibiotic courses used, respectively.

Abbreviation: BLAL, beta‐lactam allergy label.

In the context of therapeutic indications, broader‐spectrum BL antibiotics such as carbapenems (29% vs. 18%; OR 1.96, 95% CI [1.69–2.27], *p* < 0.001) and monobactams (0.9% vs. 0.1%; OR 14.85, 95% CI [5.06–43.57], *p* < 0.001) were more frequently utilized (please see Figure 2b). For prophylactic indications (Figure 2c), the use of clindamycin (66% vs. 2.4%; OR 91.02, 95% CI [69.50–119.20], *p* < 0.001) and quinolones (16% vs. 7.6%; OR 2.39, 95% CI [1.83–3.14], *p* < 0.001) was notably increased, at the expense of first generation cephalosporins, particularly cefazolin (97.7% vs. 99.4%; OR 0.21, 95% CI [0.07–0.62], *p* = 0.004). A subanalysis of patients with PenAL revealed a similar pattern in antibiotic usage (Supporting Information S1: Table E7).

The results of logistic regression analyses for each condition are detailed in Supporting Information S1: Table E6 and partially displayed in Figure 2d–f. Across all conditions, the use of penicillin and BL in general was significantly lower among patients with BLAL. Overall, the use of cephalosporins was decreased, with the exception of pneumonia patients who exhibited a pattern of reduced first‐ and increased second, third, and fourth‐generation cephalosporin usage. Meropenem and aztreonam were utilized more frequently in BLAL patients with pneumonia and those admitted to ICU.

Despite these shifts in prescribing patterns, 847 of 1394 patients with a BLAL (60.8%) received at least one BL antibiotic during admission (Supporting Information S1: Table E6). This frequent overruling of the BLAL raised the question whether next‐line antibiotic use might mediate the association between BLAL and clinical outcomes. Additional exploratory subanalyses were performed stratified by antibiotic exposure. In admissions with any non–BL exposure, BLAL was not associated with increased mortality but was associated with lower ICU admission rates and shorter LOS (Supporting Information S1: Table E4d). In admissions with BLAL, non–BL exposure was not associated with increased in‐hospital mortality, while higher 3‐month mortality was observed in the therapeutic subgroup, together with higher ICU admission rates and longer LOS (Supporting Information S1: Table E4e).

Supporting Information S1: Table E8 displays the comparative top 10 antibiotics used for each condition. For instance, in pneumonia cases, moxifloxacin (46.5%) and meropenem (29.9%) were predominantly prescribed for BLAL patients, contrasting with amoxicillin/clavulanic acid (50.5%) and piperacillin‐tazobactam (36.0%) for no‐BLAL patients.

**FIGURE 3:**
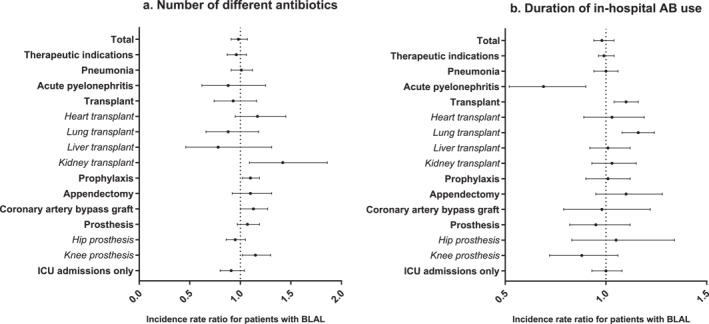
Association of reported beta‐lactam allergy labels (BLAL) with number of different antibiotics used (left) and duration of in‐hospital antibiotic use (right) in the different subpopulations. Incidence rate ratios (IRR) calculated using a multivariable Poisson model to account for differences in length of hospital stay (off‐set variable); multiple prescriptions of the same antibiotics were counted only once per admission.

There were no significant differences between BLAL and no‐BLAL patients on what concerns the number of different antibiotics used per admission (2.6 vs. 2.4; *p* = 0.7) or duration of antibiotic treatment (10.9 vs. 10.1; *p* = 0.56) as shown in Figure 3. However, the number of different antibiotics was higher in BLAL compared to non‐BLAL patients admitted for kidney transplantation (mean 4.8 (± 1.3) vs. 4.0 (± 2.3); IRR 1.42, 95% CI [1.09–1.86], *p* = 0.009) and knee prosthesis (mean 1.26 (± 0.6) vs. 1.21 (± 0.7), IRR 1.15 with 95% CI [1.02–1.3], *p* = 0.02) (Supporting Information S1: Table E9). Furthermore, BLAL were associated with extended in‐hospital antibiotic use in lung transplant patients (47.7 (± 52.77) vs. 31.4 (± 26.67) days; IRR 1.16, 95% CI [1.08–1.24], *p* < 0.001) (Figure 3b and Supporting Information S1: Table E10). In contrast, a time reduction was observed in patients with pyelonephritis (observed mean 8.6 (± 9.8) vs. 8.1 (± 2.2) days, however after correction for confounders the trend was reversed, IRR 0.69, 95% CI [0.5–0.9]; *p* = 0.009).

## 4 Discussion

European studies on the outcomes associated with BLAL are urgently needed to guide balanced delabeling protocols. Recently, two ICU‐based studies—Davis et al. (UK; 35,316 patients, 6% with BLAL) and Leone et al. (France; 7146 patients, 11% with BLAL)—specifically investigated the association with BLAL [39, 40]. Leone et al. found BLAL to be associated with a reduced mortality, while Davis et al. reported lower 28‐day mortality in patients with BLAL, especially in unplanned ICU admissions. Consistent with their findings, our results did not confirm a higher mortality associated with BLAL or PenAL. Moreover, there was no significant difference in the risk of ICU admission or increased LOS. Studies from Portugal, Spain, and the UK [25, 26, 27]—but not the Netherlands [8]—found that patients with PenAL had LOS 5.5%–8.7% longer than those without which is in contrast with our findings. Compared to US studies which indicate a 1.4 times higher ICU admission rate for patients with an antibiotic AL [5], our results suggest a lower impact on clinical outcomes. These discrepancies may be explained by differences in prescribing practices, variability in the completeness and structure of EHR data, and heterogeneity in study design—such as patient selection criteria (broad vs. condition‐specific cohorts) and covariate adjustment which can vary not only across countries but also between different clinical settings [12, 41].

In our study, antibiotic usage was significantly different in patients with a BLAL. Specifically, there was less narrow‐spectrum and more broader‐spectrum BL use. Additionally, a notable increase in the use of aztreonam was observed (OR 14.82, 95% CI [5.05–43.47]) which is in line with a US study on PenAL, reporting ORs of 0.35, 1.72 and 18.44 in PenAL [42]. Moreover, we found that BLAL was associated with increased use of non–BL antibiotics, such as clindamycin and quinolones, only partially corroborating with the findings of Davis et al. US data on PenAL also report ORs of 5.34 and 1.91 for clindamycin and quinolone use, respectively [32]. However, even when compared to other European studies [8, 10] quinolone use in our cohort was notably higher, with patients having a BLAL showing nearly a fourfold increased odds of using these antibiotics. According to the Belgian Hospitals surveillance of antimicrobial consumption (BEH‐SAC), quinolones were the second most frequently in‐hospital consumed antibiotics, accounting for 10.5%, following penicillins/betalactamase inhibitor combinations [43]. Of note, quinolones also emerged as the most commonly reported non‐BL AL in our study, with 65 (5%) in patients with BLAL, compared to only 155 (0.7%) in those without BLAL (see Supporting Information S1: Table E2).

The observed shifts in antibiotic use among patients with BLAL (Table 2 and Supporting Information S1: Table E8) partly reflect local and national guidelines recommendations [44]. Particularly, there is a notable shift toward non‐BL antibiotics, with recommendations to use moxifloxacin for community‐acquired pneumonia and clindamycin for prophylaxis during prosthesis placement. Additionally, since 2013, meropenem is considered a “safe‐to‐use” alternative, including in high‐risk PenAL patients, due to its cross‐reactivity of less than 1% and in‐hospital administration [45]. Interestingly, a significant proportion of patients with a BLAL, particularly those with unspecified PenAL, received cefazolin prophylaxis while only aligning with recent evidence [46] and post‐study guidelines [47]. This may reflect differences in adherence to or interpretation of guidelines, as well as gaps in knowledge regarding potential cross‐reactivity [35, 46, 47].

To explore whether next‐line antibiotic use might mediate the association between BLAL and clinical outcomes, we performed additional exploratory subanalyses stratified by antibiotic exposure (see Supporting Information S1: Table E4d and e). These analyses did not demonstrate a consistent association with increased mortality. While higher 3‐month mortality was observed in the therapeutic BLAL subgroup receiving non–BL antibiotics, this finding should be interpreted with caution and is most likely explained by confounding by indication, as broader or combination regimens are preferentially prescribed to patients with more severe illness. Overall, the observed associations with ICU admission and LOS were likewise most consistent with confounding by indication rather than a direct adverse effect of next‐line therapy. These findings suggest that the absence of increased mortality in our cohort is unlikely to be explained by overruling of the beta‐lactam allergy label alone.

Transplant patients are at higher risk for infections and frequent use of antibiotics [48]. Imlay et al. found that BLAL was present in 16% of solid organ transplant (SOT) and hematopoietic cell transplant (HCT) patients in the United States, mostly in lung transplant recipients, and was associated with higher use of quinolones [49]. Similarly, in our cohort, BLAL prevalence was highest among lung transplant recipients (15%), exceeding the overall prevalence in our study cohort (7.03%) and the total in‐patient population (6.01%) during the study period (see Supporting Information S1: Table E2). An Australian retrospective matched‐cohort study of 313 liver transplant recipients, found that antibiotic AL led to an increased use of cephalosporins and nitroimidazoles [50]. Similar to our findings, both studies did not observe significant associations between BLAL and mortality, ICU admissions, LOS, or readmission in multivariable analyses [49, 50].

Recent studies indicate that a thorough drug allergy workup, including detailed history, skin testing, and drug provocation tests, can successfully delabel most patients with BLAL or PenAL, potentially reducing avoidable secondary effects and supporting hospital‐wide delabeling protocols [51, 52, 53, 54, 55, 56]. Success rates vary by region, with 95%–99% in the United States and Australia [1, 32], over 90% in Belgium [57], and 77% in France [58]. However, the reported risks may not apply universally [59], emphasizing the need for localized approaches that assess risk‐benefit ratios, potentially shifting the focus to high‐risk or high‐gain populations, as well as emphasizing the value of non‐invasive delabeling protocols [60].

## 5 Limitations

Our retrospective and observational design limits the ability to establish causality between BLAL and PenAL and complications. In addition, antibiotic exposure was assessed over the entire hospital admission and could not be modeled as a time‐varying covariate. As a result, many patients received multiple antibiotic classes sequentially or concomitantly, limiting the ability to isolate the causal effect of individual treatment strategies. Furthermore, our results may be biased toward the selected conditions. Using ICD codes to identify the conditions likely underestimated their numbers [25] and only allowed for a theoretical distinction between therapeutic and prophylactic indications, which does not always reflect clinical practice. Other confounding factors, such as other antibiotic ALs, may have been overlooked although this seems to have had a minimal effect on our study population (see Supporting Information S1: Table E2). Finally, this study was conducted within a single national healthcare system, which may limit the generalizability of its findings to other European settings with different antibiotic stewardship policies and prescribing practices. On the other hand, the strengths of this study include its multivariable analysis design, the large study and well‐characterized study population with verified antibiotic use, and inclusion of high‐risk transplant and ICU patients. The potential economic burden and microbiological resistance consequences associated with BLAL were not assessed, but would provide valuable insights as previously reported [13, 61], and warrant dedicated prospective investigations (e.g., NCT06771440). Together with clinical outcomes, microbiological impact and economic burden remain important elements to co‐guide delabeling strategies. In our setting and despite altered antibiotic use, BLAL or PenAL were not associated with worse clinical outcomes in terms of LOS, mortality, or ICU admission rate. These findings may suggest that, in our European context, the clinical impact of PenAL may be less pronounced than reported elsewhere, underscoring the need for region‐specific evidence to guide delabeling strategies. Future research could focus on identifying patient subgroups that would benefit most from systematic allergy verification, as well as on evaluating the health‐economic and antimicrobial resistance benefits of structured delabeling programs across different healthcare settings.

## 6 Conclusion

Despite altered antibiotic use, BLAL or PenAL were not associated with worse clinical outcomes in terms of LOS, mortality and ICU admission rate. Together with varying BLAL and PenAL prevalences [2, 3, 4, 5, 6, 7, 8, 9, 10, 11] and the proportion of true antibiotic allergies observed in non‐EU and EU studies [1, 32, 57, 58], our findings highlight again regional differences, now in terms of clinical outcome. While non‐EU studies are often cited as an argument for hospital wide PenAL delabeling programs, the observed heterogeneity limits their transferability to a EU context.

## Author Contributions

**Liesbeth Gilissen:** conceptualization, investigation, funding acquisition, writing – original draft, methodology, validation, visualization, formal analysis, project administration, supervision, data curation, resources. **Greet Van De Sijpe:** data curation, writing – review and editing. **Annouschka Laenen:** conceptualization, methodology. **Peter Declercq:** methodology. **Dries Wets:** project administration, formal analysis. **Ileana Ghiordanescu:** writing – review and editing. **Anca Mirela Chiriac:** writing – review and editing. **Willy E. Peetermans:** writing – review and editing, conceptualization, supervision. **Paul De Munter:** conceptualization, writing – review and editing. **Isabel Spriet:** conceptualization, funding acquisition, investigation, writing – review and editing, project administration, supervision. **Rik Schrijvers:** conceptualization, funding acquisition, supervision, writing – review and editing, resources.

## Funding

This research is supported by the Research Foundation Flanders (FWO; project T003023N) and KU Leuven (C3/23/028). RS holds a senior clinical investigator fellowship from the FWO (1805523N). L.G. is a FWO junior postdoctoral fellow (12A6Q24N). IS is funded by the Clinical Research Fund of UZ Leuven.

## Conflicts of Interest

The authors declare no conflicts of interest.

## Supporting information


Supporting Information S1

## Data Availability

The data that support the findings of this study are available upon reasonable request from the corresponding author, subject to ethical approval and data protection regulations. The data are not publicly available due to privacy and ethical restrictions.
