# Supplementary material for: Clinical Outcome Associated With Beta‐Lactam Allergy Labels in Hospitalized Patients in Belgium
Source: Clin Transl Allergy. 2026 Mar 27;16(4):e70166. doi: 10.1002/clt2.70166 (PMC13140986; doi:10.1002/clt2.70166)
Supplement: Supplementary file 1 — Supporting Information S1 [file CLT2-16-e70166-s001.docx]

**ONLINE REPOSITORY**

**Allergy module at UZ Leuven – Description**

In UZ Leuven, alleged allergic reactions can be registered in the EPR system using a dedicated allergy registration and notification module (see also Gilissen et al, JACI in Practice 2021, and Van De Sijpe et al, Allergy 2022).

First, the suspected drug or drug class has to be chosen from a drop-down list, which was set up according to the Anatomical Therapeutic Chemical (ATC) classification. For BL, it is also possible to select a combination label, such as ‘BL antibiotics with exclusion of carbapenems and monobactams’, for example, in case of a history or observation of a severe reaction to penicillin/cephalosporin when it was deemed best to prohibit all BL, except those with the lowest possibility for cross-reactivity, or vice versa, ‘other BL antibiotics: carbapenems and monobactams’, when only the latter are prohibited.

Second, the onset date and symptoms of the reaction can be specified, for which distinction is made between very severe (anaphylaxis, angioedema, Stevens-Johnson/Lyell syndrome, asthma, urticaria), severe (rash, itching) or other (free text) reactions. Third, the possibility is given to specify if the reaction has been confirmed by an allergy test, (accidental) rechallenge, or because an anaphylactic shock or angioedema was observed by the clinician. In those cases, the probability of the label will automatically become ‘certain’ in the fourth and last step of the module, whereas for the others, the reporter can choose between ‘unclear’ or ‘likely’.

A registered allergy will pop-up when the EPR is consulted, as well as when an e-prescription with the respective ATC code is composed: the registration of a very severe allergic reaction generates an interruptive pop-up alert during prescribing that needs to be overruled by the prescriber in order to continue. A motivation for prescribing the drug needs to be provided. Registration of a severe reaction generates an interruptive pop-up alert, but does not need to be overruled. Registration of “other” reactions does not generate a pop-up alert but can be consulted through means of an informative ribbon bar.

Since 2016, the EPR system is also available to other UZ Leuven partner institutions in Flanders (Nexuzhealth), and to external physicians (Mynexuzhealthpro).

**Table E1.** ICD9 and ICD10 codes

| **Pneumonia** | ICD-10-CM J13 | Pneumonia due to Streptococcus pneumoniae |
| --- | --- | --- |
|  | ICD-10-CM J14 | Pneumonia due to Hemophilus influenzae |
|  | ICD-10-CM J15 | Bacterial pneumonia, not elsewhere classified |
|  | ICD-10-CM J16 | Pneumonia due to other infectious organisms, not elsewhere classified |
|  | ICD-10-CM J17 | Pneumonia in diseases classified elsewhere |
|  | ICD-10-CM J18 | Pneumonia, unspecified organism |
|  | ICD-9-CM 481 | Pneumococcal pneumonia [Streptococcus pneumoniae pneumonia] |
|  | ICD-9-CM 482 | Other bacterial pneumonia |
|  | ICD-9-CM 483 | Pneumonia due to other specified organism |
|  | ICD-9-CM 484 | Pneumonia in infectious diseases classified elsewhere |
|  | ICD-9-CM 485 | Bronchopneumonia, organism unspecified |
|  | ICD-9-CM 486 | Pneumonia, organism unspecified |
| **Acute pyelonephritis** | ICD-10-CM N10 | Acute tubulo-interstitial nephritis |
|  | ICD-9-CM 590.10 | Acute pyelonephritis without lesion of renal medullary necrosis |
|  | ICD-9-CM 590.11 | Acute pyelonephritis with lesion of renal medullary necrosis |
| **Coronary artery bypass grafting** | ICD-10-PCS 0210 | Coronary Artery, One Artery |
|  | ICD-10-PCS 0211 | Coronary Artery, Two Arteries |
|  | ICD-10-PCS 0212 | Coronary Artery, Three Arteries |
|  | ICD-10-PCS 0213 | Coronary Artery, Four or More Arteries |
|  | ICD-9-CM Procedure code 36.1 | Bypass Anastomosis For Heart Revascularization |
|  |  | Specific code36.10 Aortocoronary bypass for heart revascularization |
|  |  | Specific code 36.11 (Aorto)coronary bypass of one coronary artery |
|  |  | Specific code 36.12 (Aorto)coronary bypass of two coronary arteries |
|  |  | Specific code 36.13 (Aorto)coronary bypass of three coronary arteries |
|  |  | Specific code 36.14 (Aorto)coronary bypass of four or more coronary arteries |
|  |  | Specific code 36.15 Single internal mammary-coronary artery bypass |
|  |  | Specific code 36.16 Double internal mammary-coronary artery bypass |
|  |  | Specific code 36.17 Abdominal-coronary artery bypass |
|  |  | Specific code 36.19 Other bypass anastomosis for heart revascularization |
| **Total Knee Prosthesis** | ICD-10-PCS 0SRC | Knee Joint, Right |
|  | ICD-10-PCS 0SRD | Knee Joint, Left |
|  | ICD-9-CM Procedure 81.54 | Total knee replacement |
| **Total Hip Prosthesis** | ICD-10-PCS 0SR9 | Hip Joint, Right |
|  | ICD-10-PCS 0SRB | Hip Joint, Left |
|  | ICD-9-CM Procedure 81.51 | Total hip replacement |
| **Appendectomy** | ICD-10-PCS 0DTJ | Appendix resection |
|  | ICD-9-CM Procedure code 47.01 | Laparoscopic appendectomy |
|  | ICD-9-CM Procedure code 47.09 | Other appendectomy |
| **Transplantation** | ICD-10-PCS 02YAOZ0 | 02YA0Z0 Transplantation of Heart, Allogeneic, Open Approach |
|  | ICD-10-PCS 02YAOZ1 | 02YA0Z1 Transplantation of Heart, Syngeneic, Open Approach |
|  | ICD-10-PCS 02YAOZ2 | 02YA0Z2 Transplantation of Heart, Zooplastic, Open Approach |
|  | ICD-10-PCS 0BYK | 0BYK Lung, Right |
|  | ICD-10-PCS 0BYL | 0BYL Lung, Left |
|  | ICD-10-PCS 0BYM | 0BYM Lungs, Bilateral |
|  | ICD-10-PCS 0FY0 | 0FY00Z0 Transplantation of Liver, Allogeneic, Open Approach |
|  |  | 0FY00Z1 Transplantation of Liver, Syngeneic, Open Approach |
|  |  | 0FY00Z2 Transplantation of Liver, Zooplastic, Open Approach |
|  | ICD-10-PCS 0TY0 | 0TY0 Kidney, Right |
|  |  | 0TY00Z0 Transplantation of Right Kidney, Allogeneic, Open Approach |
|  |  | 0TY00Z1 Transplantation of Right Kidney, Syngeneic, Open Approach |
|  |  | 0TY00Z2 Transplantation of Right Kidney, Zooplastic, Open Approach |
|  | ICD-10-PCS 0TY1 | 0TY1 Kidney, Left |
|  |  | 0TY10Z0 Transplantation of Left Kidney, Allogeneic, Open Approach |
|  |  | 0TY10Z1 Transplantation of Left Kidney, Syngeneic, Open Approach |
|  |  | 0TY10Z2 Transplantation of Left Kidney, Zooplastic, Open Approach |
|  | ICD-9-CM Procedure code 37.51 | Heart transplantation |
|  | ICD-9-CM Procedure code 33.5 | Non-specific code33.5 Lung Transplant |
|  |  | Specific code33.50 Lung transplantation, not otherwise specified |
|  |  | Specific code 33.51 Unilateral lung transplantation |
|  |  | Specific code 33.52 Bilateral lung transplantation |
|  | ICD-9-CM Procedure code 33.6 | Combined heart-lung transplantation |
|  | ICD-9-CM Procedure code 50.5 | Liver Transplant |
|  |  | Specific code50.51 Auxiliary liver transplant |
|  |  | Specific code 50.59 Other transplant of liver |

**Table E2**. Numbers of patients with an antibiotic allergy label, and the respective prevalences (%), presented per AB class*^¥^*.

| **Betalactam allergy labels** | **Total study population^§^**  **(% of total, n= 21 999)** | **Therapeutic indications**  **(% of n= 15 275)** | **Prophylactic indications**  **(% of n= 7 204)** | **Total inpatients**  **(% of total, n= 273 129)** |
| --- | --- | --- | --- | --- |
| **Penicillins** | **1 201 (5.46)** | **780 (5.11)** | **443 (6.15)** | **14 858 (5.44)** |
| Penicillins general label (unspecified) | 1 113 (5.06) | 715 (4.68) | 421 (5.84) | 13 910 (5.09) |
| Penicillin G (Benzylpenicillin) | 5 (0.02) | 3 (0.02) | 3 (0.04) | 74 (0.03) |
| Flucloxacillin | 5 (0.02) | 3 (0.02) | 2 (0.03) | 56(0.02) |
| Amoxicillin (+ betalactamase-inhibitor) | 56 (0.25) | 40 (0.26) | 15 (0.21) | 740 (0.27) |
| Ampicillin | 1 (0.01) | 1 (0.01) | 0 (0.00) | 4 (<0.01) |
| Piperacillin (+ tazobactam) | 17 (0.08) | 16 (0.11) | 1 (0.01) | 60 (0.02) |
| Extended spectrum penicillins | 6 (0.03) | 5 (0.03) | 2 (0.03) | 74 (0.03) |
| **Cephalosporins** | **115 (0.52)** | **87 (0.57)** | **30 (0.42)** | **957 (0.35)** |
| Cephalosporins general label (unspecified) | 79 (0.36) | 61 (0.40) | 19 (0.26) | 693 (0.25) |
| Cephalosporins 1^st^ generation | 13 (0.06) | 9 (0.06) | 2 (0.03) | 113 (0.04) |
| Cephalosporins 2^nd^ generation | 16 (0.07) | 9 (0.06) | 6 (0.08) | 118 (0.04) |
| Cephalosporins 3^rd^ generation | 12 (0.05) | 11 (0.07) | 1 (0.01) | 51 (0.02) |
| Cephalosporins 4^th^ generation | 1 (0.01) | 1 (0.01) | **0 (0.00)** | 3 (<0.01) |
| **Carbapenems** (Meropenem) | **7 (0.03)** | **7 (0.05)** | **0 (0.00)** | **23 (0.01)** |
| **Monobactams** (Aztreonam) | **1 (0.01)** | **1 (0.01)** | **0 (0.00)** | **3 (<0.01)** |
| **Combination labels (unspecified)** | **130 (0.59)** | **108 (0.71)** | **23 (0.32)** | **105 (0.40)** |
| BL antibiotics excl. carbapenem, monobactam | 109 (0.50) | 88 (0.58) | 22 (0.31) | 1 013 (0.37) |
| Other BL antibiotics: carbapenem, monobactam | 23 (0.10) | 22 (0.15) | 1 (0.01) | 97 (0.04) |
| **Total** | **1 394 (6.34)** | **933 (6.11)** | 549 (7.62) | **16 415 (6.01)** |
| BL, beta-lactam | | | | |

| **Non-beta-lactam antibiotic allergy labels** | **Total study population**  **(% of n= 21 999, and**  **n= 1 394 *vs* n=20 605)** | | | **Therapeutic indications**  **(% of n= 15 275, and**  **n= 933 *vs* n= 14 342)** | | | **Prophylactic indications**  **(% of n= 7 204,**  **and n= 549 *vs* n= 6 655)** | | | **Total inpatients**  **(% of total, n= 273 129)** |
| --- | --- | --- | --- | --- | --- | --- | --- | --- | --- | --- |
|  | **All** | BLAL | No BLAL | **All** | BLAL | No BLAL | **All** | BLAL | No BLAL |  |
| **Quinolones** | **220 (1.00)** | 65 (4.66) | 155 (0.75) | **177 (1.16)** | 51 (5.47) | 125 (0.87) | **40 (0.56)** | 14 (2.55) | 26 (0.39) | **1 301 (0.48)** |
| **Sulfonamides/Trimethoprim** | **79 (0.36)** | 21 (1.51) | 58 (0.28) | **60 (0.39)** | 12 (1.29) | 48 (0.33) | **21 (0.29)** | 10 (1.82) | 11 (0.17) | **701 (0.26)** |
| **Macrolides** | **44 (0.20)** | 14 (1.01) | 30 (0.15) | **32 (0.21)** | 10 (1.07) | 22 (0.15) | **13 (0.18)** | 5 (0.91) | 8 (0.12) | **522 (0.19)** |
| **Nitrofuran derivatives** | **29 (0.13)** | 5 (0.35) | 24 (0.11) | **20 (0.13)** | 2 (0.21) | 18 (0.13) | **9 (0.12)** | 3 (0.55) | 6 (0.09) | **275 (0.10)** |
| **Tetracyclines** | **19 (0.09)** | 7 (0.50) | 12 (0.06) | **10 (0.07)** | 4 (0.42) | 6 (0.04) | **9 (0.12)** | 4 (0.73) | 5 (0.08) | **174 (0.06)** |
| **Lincosamides** | **20 (0.09)** | 11 (0.79) | 16 (0.08) | **16 (0.10)** | 10 (1.07) | 6 (0.04) | **5 (0.07)** | 2 (0.36) | 3 (0.05) | **96 (0.04)** |
| **Aminoglycosides**  **Glycopeptides** | **7 (0.03)**  **30 (0.14)** | 4 (0.29)  3 (0.21) | 3 (0.01)  27 (0.13) | **3 (0.01)**  **26 (0.17)** | 1 (0.11)  3 (0.32) | 2 (0.01)  23 (0.16) | **3 (0.04)**  **4 (0.06)** | 3 (0.55)  0 (0.00) | 0 (0.00)  4 (0.06) | **55 (0.02)**  X |
| **Other antibiotics** | **13 (0.06)** | 4 (0.29) | 9 (0.04) | **10 (0.07)** | 2 (0.21) | 8 (0.06) | **3 (0.04)** | 2 (0.36) | 1 (0.02) | x |
| **Total** | **418 (1.90)** | 111 (7.96) | 307 (1.49) | **321 (2.10)** | 79 (8.47) | 242 (1.69) | **91 (1.26)** | 31 (5.65) | 60 (0.90) |  |
| BLAL, beta-lactam allergy label | | | | | | | | | | |

*^¥^ As it is possible to have more than one label within the same drug class, the total number of patients per class may be less than the sum of the parts. § This cohort represents approximately 0.2% of the Belgian population, and 8% of all inpatients during the study period.*

**Table E3.** Characteristics of the patients (at first admission) per diagnosis

| **Diagnosis** | **Unique patients** | **BLAL (% of patients)** | **Patients with multiple hospitalizations**  **(% of patients)** | | | **N admissions** | **N of men**  **(% of patients)** | **Mean age**  **(±SD)** | **Mean CCI**  **(±SD)** | **ICU admissions (% of all admissions)** |
| --- | --- | --- | --- | --- | --- | --- | --- | --- | --- | --- |
|  |  |  | 2 | 3 | >3 |  |  |  |  |  |
| **Therapeutic w/o transplant** | **14 379** | **868 (6.04)** | **870 (6.05)** | **125 (0.87)** | **36 (0.25)** | **15 620** | **8438 (58.7)** | **62.3 (±24.7)** | **4.14 (±3.57)** | **4631 (29.6)** |
| **Therapeutic indications^i^** | **15 274** | **932 (6.10)** | **975 (6.38)** | **132 (0.86)** | **38 (0.25)** | **16 641** | **8 979 (58.8)** | **61.9 (**±**24.3)** | **4.11 (±3.53)** | **5 307 (31.9)** |
| Pneumonia | 13 385 | 805 (6.00) | 744 (5.56) | 107 (0.80) | 28 (0.21) | 14 433 | 8 095 (60.5) | 64.2 (±23.5) | 4.29 (±3.55) | 4 555 (31.5) |
| Pyelonephritis | 1 134 | 77 (6.79) | 70 (6.17) | 4 (0.35) | 4 (0.35) | 1 228 | 406 (35.8) | 39.2 (±30.4) | 2.50 (±3.40) | 93 (7.57) |
| Transplantation**^i^** | 1 112 | 93 (8.36) | 9 (0.81) | 0 (0) | 0 (0) | 1 121 | 672 (60.4) | 54.1 (±14.3) | 3.79 (±2.74) | 761 (67.9) |
| *Heart transplantation* | *111* | *6 (5.41)* | *1 (0.90)* | *0 (0)* | *0 (0)* | *112* | *84 (75.0)* | *46.3 (±16.7)* | *4.23 (±2.37)* | *111 (99.1)* |
| *Kidney transplantation* | *414* | *30 (7.25)* | *2 (0.48)* | *0 (0)* | *0 (0)* | *416* | *253 (61.1)* | *54.6 (±14.2)* | *2.69 (±2.29)* | *63 (15.1)* |
| *Liver transplantation* | *333* | *18 (5.41)* | *5 (1.50)* | *0 (0)* | *0 (0)* | *338* | *208 (62.5)* | *56.9 (±13.6)* | *5.90 (±2.96)* | *335 (99.1)* |
| *Lung transplantation* | *293* | *43 (14.7)* | *1 (0.54)* | *0 (0)* | *0 (0)* | *294* | *146 (49.8)* | *52.9 (±13.2)* | *3.09 (±1.99)* | *291 (99.1)* |
| **Prophylactic indications^i^** | **7203** | **549 (7.62)** | **203 (2.45)** | **7 (0.08)** | **1 (0.01)** | **7 423** | **3 936 (54.6)** | **59.5 (±20.5)** | **1.79 (±2.55)** | **2 231 (30.1)** |
| Appendectomy | 1 895 | 107 (5.65) | 1 (0.05) | 0 (0) | 0 (0) | 1 896 | 878 (46.3) | 36.4 (±21.3) | 1.23 (±2.65) | 57 (3.01) |
| Coronary artery bypass grafting | 2 433 | 146 (6.00) | 1 (0.04) | 0 (0) | 0 (0) | 2 434 | 1 878 (77.2) | 68.9 (±11.9) | 2.70 (±2.53) | 2 130 (87.5) |
| Prosthesis**^i^** | 2 905 | 239 (8.23) | 172 (5.92) | 8 (0.28) | 0 (0) | 3 093 | 1 196 (41.2) | 67.1 (±12.2) | 1.41 (±2.29) | 44 (1.42) |
| *Hip prosthesis* | *1 357* | *113 (8.33)* | *40 (2.95)* | *0 (0.00)* | *0 (0)* | *1 395* | *585 (43.0)* | *66.7* (±13.3) | *1.46 (±2.38)* | *23 (1.65)* |
| *Knee prosthesis* | *1 578* | *127 (8.05)* | *114 (7.22)* | *3 (0.19)* | *0 (0)* | *1 698* | *625 (39.6)* | *67.2* (±11.4) | *1.38 (±2.21)* | *21 (1.06)* |
| **ICU admissions** | **7 174** | **438 (6.10)** | **157 (2.19)** | **8 (0.11)** | **1 (0.01)** | **7 351** | **4 816 (67.1)** | **64.3 (±18.3)** | **3.93 (±3.20)** | **7 351 (100)** |
| **Total^i^** | **21 999** | **1 394 (6.30)** | **1 369 (6.22)** | **165 (0.75)** | **46 (0.21)** | **23 842** | **12 585 (57.2)** | **61.0 (±23.3)** | **3.34 (±3.42)** | **7 351 (30.8)** |
| (**^i^** It is possible for patients to have >1 diagnosis within the same hospitalization, therefore the total is less than the sum of the parts)  BLAL, beta-lactam allergy label; CCI, Charlson Comorbidity Index; ICU, Intensive care unit; SD, Standard deviation | | | | | | | | | | |

**Table E4a.** Multivariable regression analysis of clinical outcomes according to beta-lactam allergy label (primary analysis)(significant differences in **bold**)

| **Study population** | | **No reported BLAL** | **Reported BLAL** | **OR or HR (95% CI)** | ***p*-value** |
| --- | --- | --- | --- | --- | --- |
| Total^a^ |  |  |  |  |  |
|  | *Mortality (%)***^§^** | 1 969 (8.8) | 119 (7.7) | 0.88 (0.72-1.07) | 0.205 |
|  | *3 months mortality (%)* | 1 267 (5.7) | 89 (5.7) | 1.01 (0.80-1.27) | 0.919 |
|  | *LOS (±SD)* **^§^** | 19.92 (±31.62) | 21.57 (±39.40) | 0.86 (0.72-1.04) | 0.114 |
|  | *ICU admission (%)***^§^** | 6 901 (31.0) | 450 (29.0) | 0.92 (0.80-1.07) | 0.302 |
| Therapeutic indications^a^ | |  |  |  |  |
|  | *Mortality (%)***^§^** | 1911 (12.3) | 115 (10.9) | 0.87 (0.71-1.07) | 0.188 |
|  | *3 months mortality (%)* | 1241 (8.0) | 87 (8.3) | 1.01 (0.80-1.28) | 0.933 |
|  | *LOS (±SD)* **^§^** | 24.08 (**±**36.96) | 26.80 (**±**46.25) | 0.86 (0.71-1.03) | 0.105 |
|  | *ICU admission (%)***^§^** | 4980 (31.9) | 327 (31.1) | 0.94 (0.81-1.09) | 0.411 |
| Therapeutic indications w/o transplant^a^**^§^** | |  |  |  |  |
|  | *Mortality (%)* | 1881 (12.8) | 113 (11.6) | 0.88 (0.71-1.08) | 0.205 |
|  | *3 months mortality (%)* | 1236 (8.4) | 85 (8.7) | 0.99 (0.78-1.25) | 0.923 |
|  | *LOS (±SD)* | 23.56 (**±**36.06) | 25.95 (**±**46.19) | 0.85 (0.71-1.03) | 0.093 |
|  | *ICU admission (%)* | 4556 (29.7) | 275 (28.2) | 0.98 (0.84-1.14) | 0.781 |
| Prophylactic indications^a^ | |  |  |  |  |
|  | *Mortality (%)* | 70 (1.1) | 5 (1.0) | 1.03 (0.41-2.61) | 0.954 |
|  | *3 months mortality (%)* | 34 (0.5) | 2 (0.4) | 0.85 (0.20-3.60) | 0.819 |
|  | *LOS (±SD)* | 10.95 (±15.90) | 11.02 (**±**12.97) | 0.96 (0.86-1.05) | 0.336 |
|  | *ICU admission (%)* | 2099 (30.3) | 132 (25.8) | 0.85 (0.55-1.30) | 0.443 |
| Appendectomy | |  |  |  |  |
|  | *Mortality (%)* | 8 (0.4) | 0 (0) | NA | 1.000**^§§^** |
|  | *3 months mortality (%)* | 4 (0.2) | 0 (0) | NA | 1.000**^§§^** |
|  | *LOS (±SD)* | 6.63 (±12.31) | 7.39 (±10.59) | 0.986 ( 0.81-1.20) | 0.886 |
|  | *ICU admission (%)* | 52 (2.9) | 5 (4.7) | 1.65 (0.62-4.39) | 0.316 |
| Coronary artery bypass | |  |  |  |  |
|  | *Mortality (%)* | 60 (2.6) | 5 (3.4) | 1.21 (0.47-3.11) | 0.692 |
|  | *3 months mortality (%)* | 22 (1) | 0 (0) | NA | 0.638**^§§^** |
|  | *LOS (±SD)* | 15.61 (19.35) | 15.88 (±19.53) | 1.08 (0.91-1.29) | 0.360 |
|  | *ICU admission (%)* | 2 005 (87.6) | 125 (85.6) | 0.76 (0.46-1.26) | 0.288 |
| Prosthesis^b^ | |  |  |  |  |
|  | *Mortality (%)***^§^** | 2 (0.1) | 0 (0) | NA | 1.000**^§§^** |
|  | *3 months mortality (%)* | 8 (0.3) | 20 (0.8) | 2.68 (0.48-15.10) | 0.264 |
|  | *LOS (±SD)* | 9.9 (±13.72) | 9.77 (±7.2) | 0.93 (0.82-1.06) | 0.262 |
|  | *ICU admission (%)***^§^** | 42 (1.5) | 2 (0.8) | 0.44 (0.10-1.98) | 0.287 |
| Hip prosthesis | |  |  |  |  |
|  | *Mortality (%)* | 2 (0.2) | 0 | NA | 1.000**^§§^** |
|  | *3 months mortality (%)* | 6 (0.5) | 2 (1.7) | 4.36 (0.75-25.52) | 0.102 |
|  | *LOS (±SD)* | 10.49 (12.93) | 10.21 (±6.99) | 0.95 (0.85-1.05) | 0.312 |
|  | *ICU admission (%)* | 22 (1.7) | 1 (0.3) | 0.46 (0.06-3.54) | 0.455 |
| Knee prosthesis**^§^** | |  |  |  |  |
|  | *Mortality (%)***^§^** | 0 (0) | 0 (0) | NA | NA |
|  | *3 months mortality (%)***^§^** | 2 (0.1) | 0 (0) | NA | 1.000**^§§^** |
|  | *LOS (±SD)* | 9.42 (±14.32) | 9.4 (±7.38) | 0.89 (0.75-1.06) | 0.183 |
|  | *ICU admission (%)***^§^** | 20 (1.3) | 1 (0.7) | 0.63 (0.08-4.79) | 0.656 |
| Pneumonia**^§^** | |  |  |  |  |
|  | *Mortality (%)* | 1864 (13.8) | 111 (12.4) | 0.87 (0.71-1.07) | 0.199 |
|  | *3 months mortality (%)* | 1205 (8.9) | 81 (9) | 0.98 (0.62-1.22) | 0.884 |
|  | *LOS (±SD)* | 24.47 (±36.77) | 26.36 (±46.28) | 0.85 (0.71-1.03) | 0.102 |
|  | *ICU admission (%)* | 4 286 (31.7) | 265 (29.5) | 0.96 (0.69-1.09) | 0.559 |
| Acute pyelonephritis**^§^** | |  |  |  |  |
|  | *Mortality (%)* | 22 (1.9) | 2 (2.4) | 0.90 (0.21-3.86) | 0.889 |
|  | *3 months mortality (%)* | 33 (2.9) | 5 (6.2) | 1.73 (0.61-4.92) | 0.302 |
|  | *LOS (±SD)* | 13.37 (±24.28) | 21.67 (±44.64) | 0.48 (0.16-1.41) | 0.182 |
|  | *ICU admission (%)* | 82 (7.2) | 11 (13.3) | 1.60 (0.80-3.19) | 0.186 |
| Transplant^c^ | |  |  |  |  |
|  | *Mortality (%)* | 35 (3.4) | 4 (4.3) | 1.11 (0.37-3.29) | 0.850 |
|  | *3 months mortality (%)* | 7 (0.7) | 2 (2.2) | 2.30 (0.29-18.03) | 0.430 |
|  | *LOS (±SD)* | 35.02 (±38.77) | 41.12 (±49.44) | 1.01 (0.81-1.26) | 0.928 |
|  | *ICU admission (%)* | 695 (67.6) | 66 (71) | 0.62 (0.17-2.28) | 0.469 |
| Heart transplant | |  |  |  |  |
|  | *Mortality (%)* | 7 (6.06) | 0 (0) | NA | 1.000**^§§^** |
|  | *3 months mortality (%)* | 0 (0) | 0 (0) | NA | NA |
|  | *LOS (±SD)* | 44 (±63.82) | 33.83 (±13.83) | 0.87 (0.55-1.40) | 0.87 |
|  | *ICU admission (%)* | 10 (99.1) | 6 (100) | NA | 1.000**^§§^** |
| Lung transplant | |  |  |  |  |
|  | *Mortality (%)* | 12 (4.8) | 4 (9.3) | 2.10 (0.62-7.06) | 0.233 |
|  | *3 months mortality (%)* | 3 (1.3) | 1 (2.6) | 1.70 (0.16-17.89) | 0.659 |
|  | *LOS (±SD)* | 41.96 (±36.44) | 54.74 (±57.44) | 0.82 (0.58-1.15) | 0.247 |
|  | *ICU admission (%)* | 248 (98.8) | 43 (100) | NA | 1.000**^§§^** |
| Liver transplant | |  |  |  |  |
|  | *Mortality (%)* | 13 (4.1) | 0 (0) | NA | 1.000**^§§^** |
|  | *3 months mortality (%)* | 2 (0.7) | 1 (5.6) | 14.24 (0.09-2207.57) | 0.302 |
|  | *LOS (±SD)* | 42.78 (±47.37) | 45.67 (±58.61) | 1.06 (0.66-1.71) | 0.819 |
|  | *ICU admission (%)* | 320 (100) | 18 (100) | NA | 1.000**^§§^** |
| Kidney transplant | |  |  |  |  |
|  | *Mortality (%)* | 5 (1.3) | 0 (0) | NA | 1.000**^§§^** |
|  | *3 months mortality (%)* | 3 (0.8) | 1 (3.3) | 3.20 (0.01-805.89) | 0.680 |
|  | *LOS (±SD)* | 25.66 (±30.75) | 21.57 (±19.03) | 1.41 (0.96-2.07) | 0.079 |
|  | *ICU admission (%)* | 60 (15.5) | 3 (10) | 0.46 (0.13-1.61) | 0.177 |
| ICU admissions only^a^ | |  |  |  |  |
|  | *Mortality (%)* | 1087 (15.8) | 76 (16.9) | 1.12 (0.85-1.47) | 0.414 |
|  | *3 months mortality (%)* | 322 (4.7) | 20 (4.5) | 0.91 (0.56-1.45) | 0.677 |
|  | *LOS (±SD)*^¥^ | 32.94 (±46.32) | 40.32 (±65.61) | 0.95 (0.85-1.05) | 0.294 |
| BLAL, beta-lactam allergy label; CI, confidence interval; ICU, intensive care unit; LOS, length of hospital stay; NA, not available (amount of subjects equals zero in at least one of the groups); OR, odds ratio; HR, hazard ratio; SD, standard deviation  ^a^ diagnoses, ^b^ type of prosthesis (hip or knee), and ^c^ type of transplant added to the model as confounders  ^§^ GEE procedure used; ^§§^ 2-sided p-value by fisher’s exact test  ^¥^ Duration of the total hospital admission (i.e., including days at other departments during the same admission) | | | | | |

**Table E4b.** Sensitivity analysis restricted to first hospitalizations only

(significant differences in **bold**)

| **Study population** | | **No reported BLAL** | **Reported BLAL** | **OR or HR (95% CI)** | ***p*-value** |
| --- | --- | --- | --- | --- | --- |
| Total^a^ |  |  |  |  |  |
|  | *Mortality (%)* | 1860 (9.03) | 107 (7.68) | 0.87 (0.71-1.08) | 0.208 |
|  | *3 months mortality (%)* | 1166 (5.66) | 78 (5.6) | 1.01 (0.79-1.29) | 0.942 |
|  | *LOS (±SD)* | 19.88 (31.69) | 21.53 (40.29) | 0.98 (0.92-1.03) | 0.413 |
|  | *ICU admission (%)* | 6505 (31.57) | 416 (29.84) | 0.93 (0.8-1.09) | 0.37 |
| Therapeutic indications^a^ | |  |  |  |  |
|  | *Mortality (%)* | 1815 (12.66) | 105 (11.28) | 0.88 (0.71-1.08) | 0.221 |
|  | *3 months mortality (%)* | 1162 (8.1) | 77 (8.27) | 0.99 (0.78-1.27) | 0.964 |
|  | *LOS (±SD)* | 24.28 (36.17) | 27.09 (47.7) | 0.99 (0.92-1.06) | 0.777 |
|  | *ICU admission (%)* | 4677 (32.61) | 303 (32.51) | 0.96 (0.82-1.12) | 0.608 |
| Prophylactic indications^a^ | |  |  |  |  |
|  | *Mortality (%)* | 69 (1.03) | 5 (1.02) | 1.03 (0.4-2.62) | 0.952 |
|  | *3 months mortality (%)* | 32 (0.48) | 1 (0.20) | 0.46 (0.06-3.43) | 0.449 |
|  | *LOS (±SD)* | 11.01 (±16.08) | 11.16 (±13.19) | 0.95 (0.87-1.04) | 0.296 |
|  | *ICU admission (%)* | 2084 (31.04) | 131 (26.79) | 0.82 (0.53-1.27) | 0.38 |
| BLAL, beta-lactam allergy label; CI, confidence interval; ICU, intensive care unit; LOS, length of hospital stay; OR, odds ratio; HR, hazard ratio; SD, standard deviation | | | | | |
| ^a^ diagnoses added to the model as confounders | | | | | |

**Table E4c.** Sensitivity analysis restricted to patients with BLAL already before hospitalization

(413 patients labelled during hospitalization excluded; significant differences in **bold**)

| **Study population** | | **No reported BLAL** | **Reported BLAL** | **OR or HR (95% CI)** | ***p*-value** |
| --- | --- | --- | --- | --- | --- |
| Total^a^ |  |  |  |  |  |
|  | *Mortality (%)***^§^** | 1 969 (8.8) | 82 (7.2) | 0.81 (0.64-1.04) | 0.205 |
|  | *3 months mortality (%)* | 1 267 (5.7) | 73 (6.4) | 1.11 (0.86-1.43) | 0.919 |
|  | *LOS (±SD)* **^§^** | 19.92 (±31.62) | 19.09 (±25.61) | 0.94 (0.75-1.17) | 0.561 |
|  | *ICU admission (%)***^§^** | 6 901 (31.0) | 285 (25.0) | **0.78 (0.65-0.93)** | **0.006** |
| Therapeutic indications^a^ | |  |  |  |  |
|  | *Mortality (%)***^§^** | 1911 (12.3) | 79 (10.3) | 0.87 (0.71-1.07) | 0.188 |
|  | *3 months mortality (%)* | 1241 (8.0) | 72 (9.4) | 1.01 (0.80-1.28) | 0.933 |
|  | *LOS (±SD)* **^§^** | 24.08 (±36.96) | 22.94 (±29.09) | 0.93 (0.74-1.16) | 0.496 |
|  | *ICU admission (%)***^§^** | 4980 (31.9) | 221 (28.8) | 0.94 (0.81-1.09) | 0.411 |
| Prophylactic indications^a^ | |  |  |  |  |
|  | *Mortality (%)* | 70 (1.1) | 3 (0.8) | 0.997 (0.30-3.30) | 0.996 |
|  | *3 months mortality (%)* | 34 (0.5) | 1 (0.3) | 0.54 (0.07-4.07) | 0.552 |
|  | *LOS (±SD)* | 10.95 (±15.90) | 11.73 (±14.62) | 0.73 (0.42-1.27) | 0.269 |
|  | *ICU admission (%)* | 2099 (30.3) | 69 (18.2) | 0.91 (0.82-1.01) | 0.074 |
| BLAL, beta-lactam allergy label; CI, confidence interval; ICU, intensive care unit; LOS, length of hospital stay; OR, odds ratio; HR, hazard ratio; SD, standard deviation  ^a^ diagnoses added to the model as confounders  ^§^ GEE procedure used | | | | | |

**Table E4d.** Multivariable regression analysis in patients who used next-line antibiotics (any non-beta-lactam antibiotic (BL AB) exposure)(exploratory subanalysis)(significant differences in **bold**)

| **Population: any non-BL AB use** | | **No reported BLAL** | **Reported BLAL** | **OR or HR (95% CI)** | ***p*-value** |
| --- | --- | --- | --- | --- | --- |
| Total^a^ | n = 12612 | n = 11300 | n = 1312 |  |  |
|  | *Mortality (%)***^§^** | 1 401 (12.4) | 101 (7.7) | 0.64 (0.51-0.79) | **<0.001** |
|  | *3 months mortality (%)* | 687 (6.1) | 83 (6.3) | 1.12 (0.88-1.43) | 0.370 |
|  | *LOS (±SD)* **^§^** | 27.21 (±41.48) | 22.99 (±42.36) | 1.24 (1.17-1.32) | **<0.001** |
|  | *ICU admission (%)***^§^** | 4 078 (36.1) | 380 (29.0) | 0.69 (0.58-0.80) | **<0.001** |
| Therapeutic indications^a^  n = 10088 | | n = 9 163 | n = 925 |  |  |
|  | *Mortality (%)* | 1365 (14.9) | 98 (10.6) | 1.02 (1.02-1.03) | **<0.001** |
|  | *3 months mortality (%)* | 671 (7.3) | 83 (9.0) | 1.17 (0.92-1.50) | 0.206 |
|  | *LOS (±SD)* | 30.74 (±44.07) | 28.19 (±48.87) | 1.18 (1.10-1.27) | **<0.001** |
|  | *ICU admission (%)* | 3 671 (40.1) | 304 (32.9) | 0.72 (0.62-0.85) | **<0.001** |
| Prophylactic indications^a^  n = 2 651 | | n = 2 250 | n = 402 |  |  |
|  | *Mortality (%)* | 51 (2.3) | 5 (1.2) | 1.02 (0.99-1.04) | 0.122 |
|  | *3 months mortality (%)* | 21 (0.9) | 1 (0.2) | 0.19 (0.02-1.50) | 0.114 |
|  | *LOS (±SD)* | 13.72 (±25.381) | 11.19 (±14.05) | 1.36 (1.22-1.53) | **<0.001** |
|  | *ICU admission (%)* | 503 (22.4) | 87 (21.6) | 0.48 (0.27-0.83) | 0.009 |
| BLAL, beta-lactam allergy label; CI, confidence interval; ICU, intensive care unit; LOS, length of hospital stay; OR, odds ratio; HR, hazard ratio; SD, standard deviation. ^a^ diagnoses added to the model as confounder; ^§^ GEE procedure used | | | | | |

**Table E4e.** Multivariable regression analysis in patients with a beta-lactam allergy label (BLAL), stratified by non-beta-lactam antibiotic exposure (exploratory subanalysis)(significant differences in **bold**)

| **Population: reported BLAL** | | **No non-BL AB use** | **Any non-BL AB use** | **OR or HR (95% CI)** | ***p*-value** |
| --- | --- | --- | --- | --- | --- |
| Total^a^ | n = 1552 | n = 240 | n =  1312 |  |  |
|  | *Mortality (%)* | 18 (7.5) | 101 (7.7) | 0.90 (0.52-1.55) | 0.701 |
|  | *3 months mortality (%)* | 6 (2.5) | 83 (6.3) | 2.17 (0.91-5.30) | 0.081 |
|  | *LOS (±SD)* | 13.83 (±12.62) | 22.98 (±42.35) | 0.81 (0.70-0.95) | **0.009** |
|  | *ICU admission (%)* | 70 (29.2) | 380 (29.0) | 1.76 (1.15-2.68) | **0.008** |
| Therapeutic indications^a^  n = 1 053 | | n = 123 | n = 930 |  |  |
|  | *Mortality (%)* | 17 (13.9) | 98 (10.5) | 0.83 (0.47-1.45) | 0.503 |
|  | *3 months mortality (%)* | 4 (3.3) | 83 (8.9) | 3.28 (1.16-9.30) | **0.025** |
|  | *LOS (±SD)* | 16.78 (±15.29) | 28.11 (±48.75) | 0.76 (0.61-0.93) | **0.009** |
|  | *ICU admission (%)* | 25 (20.3) | 302 (32.5) | 1.60 (1.01-2.56) | **0.048** |
| Prophylactic indications^a^  n = 511 | | n = 111 | n = 400 |  |  |
|  | *Mortality (%)* | 1 (0.9) | 4 (1.0) | 1.65 (0.16-17.17) | 0.674 |
|  | *3 months mortality (%)* | 1 (0.9) | 1 (0.3) | 0.01 (0.01-42.80) | 0.359 |
|  | *LOS (±SD)* | 10.69 (±8.14) | 11.11 (±14.02) | 0.88 (0.70-1.10) | 0.266 |
|  | *ICU admission (%)* | 47 (42.8) | 85 (21.3) | 1.15 (0.60-4.02) | 0.364 |
| BLAL, beta-lactam allergy label; CI, confidence interval; ICU, intensive care unit; LOS, length of hospital stay; OR, odds ratio; HR, hazard ratio; SD, standard deviation. ^a^ diagnoses added to the model as confounder | | | | | |

**Table E5.** Results of the multivariable regression analysis for PenAL (significant differences in **bold**)

| **Study population** | | **No reported PenAL** | **Reported PenAL** | **OR or HR (95% CI)** | ***p*-value** |
| --- | --- | --- | --- | --- | --- |
| Total^a^ |  |  |  |  |  |
|  | *Mortality (%) ^§^* | 1987 (8.8) | 101 (7.6) | 0.88 (0.71-1.09) | 0.231 |
|  | *3 months mortality (%)* | 1275 (5.7) | 81 (6.1) | 1.10 (0.86-1.4) | 0.437 |
|  | *LOS (±SD)* **^§^** | 19.97 (±31.74) | 21.1 (±39.02) | 0.98 (0.93-1.04) | 0.545 |
|  | *ICU admission (%)***^§^** | 6959 (30.9) | 392 (29.6) | 0.95 (0.81-1.11) | 0.514 |
| Therapeutic indications^a^ | |  |  |  |  |
|  | *Mortality (%)^§^* | 1928 (12.2) | 98 (11.2) | 0.88 (0.7-1.09) | 0.243 |
|  | *3 months mortality (%)* | 1249 (7.9) | 79 (9.03) | 1.1 (0.86-1.4) | 0.448 |
|  | *LOS (±SD)* | 24.13 (36.09) | 26.47 (46.36) | 1 (0.93-1.07) | 0.935 |
|  | *ICU admission (%)^§^* | 5030 (31.9) | 277 (31.6) | 0.96 (0.82-1.14) | 0.665 |
| Prophylactic indications^a^ | |  |  |  |  |
|  | *Mortality (%)* | 71 (1) | 4 (0.9) | 0.86 (0.31-2.41) | 0.773 |
|  | *3 months mortality (%)* | 34 (0.5) | 2 (0.4) | 0.91 (0.21-3.87) | 0.897 |
|  | *LOS (±SD)* | 10.94 (10.94) | 11.16 (11.16) | 0.97 (0.88-1.06) | 0.469 |
|  | *ICU admission (%)* | 2110 (30.3) | 121 (26.3) | 0.77 (0.5-1.19) | 0.242 |
| PenAL, penicillin allergy label; CI, confidence interval; ICU, intensive care unit; LOS, length of hospital stay; OR, odds ratio; HR, hazard ratio; SD, standard deviation  ^a^ diagnoses added to the model as confounders  ^§^ GEE procedure used | | | | | |

**Table E6.** Associations of a reported BLAL with antibiotic use per study population with OR or β, as calculated via multiple regression (significant differences between those with and without a reported BLAL in **bold)**

| **Study population** | **No reported BLAL** | **Reported BLAL** | **OR or β (95% CI)** | ***p*-value** |
| --- | --- | --- | --- | --- |
| Total study population^a^  N admissions (n=23 842)  Antibiotic use  BL AB (%)**^§^**  *Narrow spectrum BL***^§^**^¥^  *Broad spectrum BL***^§^**^¥^  Penicillin AB (%)**^§^**  Amoxicillin/clavulanic acid**^§^**  Piperacillin/tazobactam**^§^**  Flucloxacillin**^§^**  Cephalosporin AB (%)**^§^**  *First generation***^§^**  *Second generation***^§^**  *Third generation***^§^**  *Fourth generation*  Carbapenem AB (%)**^§^**  Monobactam AB (%)  Tetracycline AB (%)  Quinolone AB (%)**^§^**  Aminoglycoside AB (%)**^§^**  Macrolide AB (%)**^§^**  Lincosamide AB (%)**^§^**  Clindamycin**^§^**  Glycopeptides (%)**^§^**  Vancomycin**^§^**  Others (%) | 22290 (93.5)  21 059 (94.5)  17 248 (81.9)  9 490 (45.1)  13 144 (59.0)  8 056 (36.1)  5 726 (25.7)  720 (3.2)  12 352 (55.4)  9 071 (73.4)  402 (3.3)  3 767 (30.5)  111 (0.9)  2 856 (12.8)  8 (0.04)  161 (0.7)  4 731 (21.2)  2 887 (13)  2 579 (11.6)  626 (2.8)  624 (2.8)  2 668 (12.0)  2 539 (11.4)  4 427 (19.5) | 1552 (6.5)  922 (59.4)  496 (53.8)  622 (67.5)  391 (25.2)  159 (10.2)  247 (15.9)  26 (1.7)  551 (35.5)  304 (55.2)  32 (5.8)  269 (48.8)  17 (3.1)  320 (20.6)  9 (0.6)  20 (1.3)  714 (46.0)  269 (17.3)  208 (13.4)  501 (32.3)  501 (32.3)  238 (15.3)  228 (14.7)  398 (25.1) | **0.08 (0.07-0.09)**  **0.21 (0.18-0.25)**  **2.88 (2.44-3.4)**  **0.15 (0.13-0.17)**  **0.17 (0.14-0.20)**  **0.51 (0.44-0.60)**  **0.52 (0.34-0.78)**  **0.21 (0.17-0.27)**  **0.37 (0.28-0.51)**  1.3 (0.88-1.92)  **2.14 (1.66-2.76)**  **2.48 (1.46-4.22)**  **1.94 (1.68-2.24)**  **14.82 (5.05-43.47)**  **1.68 (1.04-2.73)**  **3.57 (3.18-4.02)**  **1.42 (1.21-1.66)**  1.13 (0.96-1.34)  **17.24 (15.03-19.79)**  **17.31 (15.08-19.86)**  **1.43 (1.23-1.67)**  **1.43 (1.22-1.67)**  **1.63 (1.39-1.90)** | **<0.001**  **<0.001**  **<0.001**  **<0.001**  **<0.001**  **<0.001**  **0.001**  **<0.001**  **<0.001**  0.193  **<0.001**  **<0.001**  **<0.001**  **<0.001**  **0.034**  **<0.001**  **<0.001**  0.148  **<0.001**  **<0.001**  **<0.001**  **<0.001**  **<0.001** |
| Therapeutic indications^a^  N admissions (n= 16 641)  Antibiotic use  BL AB (%)**^§^**  *Narrow spectrum BL***^§^**^¥^  *Broad spectrum BL***^§^**^¥^  Penicillin AB (%)**^§^**  Amoxicillin/clavulanic acid**^§^**  Piperacillin/tazobactam**^§^**  Flucloxacillin  Cephalosporin AB (%)**^§^**  *First generation***^§^**  *Second generation***^§^**  *Third generation***^§^**  *Fourth generation***^§^**  Carbapenem AB (%)**^§^**  Monobactam AB (%)  Tetracycline AB (%)  Quinolone AB (%)**^§^**  Aminoglycoside AB (%)**^§^**  Macrolide AB**^§^**  Lincosamide AB (%) **^§^**  Clindamycin**^§^**  Glycopeptides (%)**^§^**  Vancomycin**^§^**  Others (%)**^§^** | 15588 (93.7)  14 427 (92.6)  10 664 (73.9)  8 893 (61.6)  12 111 (77.7)  7 396 (47.4)  5 298 (34.0 )  636 (4.1)  5 954 (38.2)  2 699 (45.3)  382 (6.4)  3 592(60.3)  107 (1.8)  2 747 (17.6)  8 (0.1)  143 (0.9)  4 259 (27.3)  2 751 (17.6)  2 527 (16.2)  465(3)  463 (3)  2 463 (15.8)  2 334 (15)  3 850 (24.7) | 1053 (6.3)  694 (65.9)  279 (40.2)  592 (85.3)  364 (34.6)  148 (14.1)  232 (22)  24 (2.3)  342 (32.5)  99 (28.9)  29 (8.5)  258 (75.4)  17 (5)  308 (29.2)  9 (0.9)  19 (1.8)  641 (60.9)  258 (24.5)  197 (18.7)  170 (16.1)  170 (16.1)  214 (20.3)  204 (19.4)  348 (33.0) | **0.15 (0.13-0.18)**  **0.22 (0.19-0.26)**  **3.57 (2.85-4.47)**  **0.14 (0.12-0.16)**  **0.16 (0.13-0.2)**  **0.52 (0.44-0.61)**  **0.56 (0.37-0.85)**  **0.74 (0.63-0.87)**  **0.4 (0.3-0.53)**  1.23 (0.83-1.84)  **2.22 (1.68-2.94)**  **2.56 (1.51-4.35)**  **1.96 (1.69-2.27)**  **14.85 (5.06-43.57)**  **1.85 (1.13-3.04)**  **4.01 (3.51-4.59)**  **1.46 (1.24-1.72)**  1.09 (0.91-1.29)  **6.45 (5.34-7.81)**  **6.48 (5.36-7.84)**  **1.42 (1.21-1.67)**  **1.41 (1.2-1.67)**  **1.63 (1.38-1.89)** | **<0.001**  **<0.001**  **<0.001**  **<0.001**  **<0.001**  **<0.001**  **0.006**  **<0.001**  **<0.001**  0.305  **<0.001**  **0.001**  **<0.001**  **<0.001**  **0.015**  **<0.001**  **<0.001**  0.353  **<0.001**  **<0.001**  **<0.001**  **<0.001**  **<0.001** |
| Therapeutic w/o transplant^a^  N admissions (n= 15620)  Antibiotic use  BL AB (%)**^§^**  *Narrow spectrum BL***^§^**^¥^  *Broad spectrum BL***^§^**^¥^  Penicillin AB (%)**^§^**  Amoxicillin/clavulanic acid**^§^**  Piperacillin/tazobactam**^§^**  Flucloxacillin  Cephalosporin AB (%)**^§^**  *First generation***^§^**  *Second generation***^§^**  *Third generation***^§^**  *Fourth generation***^§^**  Carbapenem AB (%)**^§^**  Monobactam AB (%)  Tetracycline AB (%)  Quinolone AB (%)**^§^**  Aminoglycoside AB (%)**^§^**  Macrolide AB**^§^**  Lincosamide AB (%) **^§^**  Clindamycin**^§^**  Glycopeptides (%)**^§^**  Vancomycin**^§^**  Others (%)**^§^** | 14 644 (93.8)  13490 (92.1)  9765 (72.4)  8302 (61.5)  11471 (78.3)  7148 (48.8)  5016 (34.3)  546 (3.7)  5056 (34.5)  2075 (41)  351 (6.9)  3226 (63.8)  102 (2)  2505 (17.1)  5 (0)  128 (0.9)  4048 (27.6)  1998 (13.6)  2345 (16)  432 (3)  431 (2.9)  2163 (14.8)  2034 (13.9)  2911 (19.9) | 976 (6.25)  645 (66.1)  247 (38.3)  549 (85.1)  335 (34.3)  138 (14.1)  211 (21.6)  20 (2)  308 (31.6)  76 (24.7)  28 (9.1)  240 (77.9)  15 (4.9)  279 (28.6)  6 (0.6)  15 (1.5)  600 (61.5)  181 (18.5)  165 (16.9)  134 (13.7)  134 (13.7)  181 (18.5)  171 (17.5)  272 (27.9) | **0.17 (0.15-0.2)**  **0.24 (0.21-0.29)**  **3.61 (2.87-4.55)**  **0.14 (0.12-0.17)**  **0.7 (0.68-0.72)**  **0.51 (0.44-0.61)**  **0.61 (0.39-0.96)**  0.92 (0.8-1.07)  **0.47 (0.36-0.62)**  1.26 (0.84-1.89)  **2.07 (1.55-2.75)**  **2.3 (1.31-4.03)**  **2.07 (1.78-2.41)**  **25.22 (7.05-90.27)**  **1.86 (1.08-3.20)**  **3.98 (3.47-4.57)**  **1.41 (1.19-1.68)**  1.09 (0.91-1.31)  **5.34 (4.33-6.59)**  **5.35 (4.34-6.61)**  **1.41 (1.19-1.68)**  **1.41 (1.18-1.68)**  **1.63 (1.40-1.90)** | **<0.001**  **<0.001**  **<0.001**  **<0.001**  **<0.001**  **<0.001**  **0.033**  0.277  **<0.001**  0.255  **<0.001**  **0.004**  **<0.001**  **<0.001**  **0.026**  **<0.001**  **<0.001**  0.328  **<0.001**  **<0.001**  **<0.001**  **<0.001**  **<0.001** |
| Pneumonia**^§^**  N admissions (n= 14 433)  Antibiotic use  BL AB (%)  *Narrow spectrum BL*^¥^  *Broad spectrum BL*^¥^  Penicillin AB (%)  Amoxicillin/clavulanic acid  Piperacillin/tazobactam  Flucloxacillin  Cephalosporin AB (%)  *First generation*  *Second generation*  *Third generation*  *Fourth generation*  Carbapenem AB (%)  Monobactam AB (%)  Tetracycline AB (%)  Quinolone AB (%)  Aminoglycoside AB (%)  Macrolide AB (%)  Lincosamide AB (%)  Clindamycin  Glycopeptides (%)  Vancomycin  Others (%) | 13536 (93.8)  12564(92.8)  17600(82.1)  9785(45.7)  10838(80.1)  6843(50.6)  4873(36)  527(3.9)  4436(32.8)  9350(73.6)  428(3.4)  3902(30.7)  116(0.9)  2421(17.9)  5(0)  124(0.9)  3571(26.4)  1623(12)  2338(17.3)  417(3.1)  416(3.1)  2082(15.4)  1953(14.4)  1691 (12.5) | 897 (6.2)  596(66.4)  519(54.5)  651(68.3)  306(34.1)  128(14.3)  202(22.5)  19(2.1)  280(31.2)  324(56.3)  33(5.7)  284(49.3)  17(3)  269(30)  5(0.6)  14(1.6)  547(61)  132(14.7)  164(18.3)  123(13.7)  123(13.7)  169(18.8)  159(17.7)  180 (20.1) | **0.16 (0.14-0.18)**  **0.27 (0.23-0.3)**  **2.6 (2.25-3)**  **0.13 (0.11-0.15)**  **0.16 (0.13-0.19)**  **0.5 (0.43-0.59)**  **0.59 (0.37-0.95)**  0.96 (0.83-1.11)  **0.48 (0.4-0.57)**  **1.58 (1.09-2.28)**  **2.19 (1.83-2.61)**  **2.9 (1.72-4.89)**  **2.07 (1.78-2.41)**  **19.95 (5.34-74.46)**  **1.79 (1.02-3.13)**  **4.21 (3.66-4.84)**  **1.29 (1.06-1.56)**  1.11 (0.93-1.32)  **5.12 (4.12-6.35)**  **5.13 (4.13-6.37)**  **1.34 (1.13-1.6)**  **1.34 (1.12-1.6)**  **1.71 (1.44-2.03)** | **<0.001**  **<0.001**  **<0.001**  **<0.001**  **<0.001**  **<0.001**  **0.028**  0.594  **<0.001**  **0.015**  **<0.001**  **<0.001**  **<0.001**  **<0.001**  **0.043**  **<0.001**  **0.01**  0.264  **<0.001**  **<0.001**  **0.001**  **0.001**  **<0.001** |
| Transplantation^c^  N admissions (n= 1 121)  Antibiotic use  BL AB (%)  *Narrow spectrum BL*^¥^  *Broad spectrum BL*^¥^  Penicillin AB (%)  Amoxicillin/clavulanic acid  Piperacillin/tazobactam  Flucloxacillin  Cephalosporin AB (%)  *First generation*  *Second generation*  *Third generation*  *Fourth generation*  Carbapenem AB (%)  Monobactam AB (%)  Tetracycline AB (%)  Quinolone AB (%)  Aminoglycoside AB (%)  Macrolide AB (%)  Lincosamide AB (%)  Clindamycin  Glycopeptides (%)  Vancomycin  Others (%) | 1028 (91.7)  1 021 (99.3)  *982 (96.2)*  *673 (65.9)*  715 (69.6)  286 (27.8)  337 ( (32.8)  104 (10.1)  981 (95.4)  *682 (69.5)*  *38 (3.9)*  *412 (42.0)*  *6 (0.6)*  292 (28.4)  5 (0.5)  18 (1.8)  244 (23.7)  829 (80.6)  215 (20.9)  35 (3.4)  34 (3.3)  339 (33.0)  339 (33.0)  1 022 (99.4) | 93 (8.30)  65 (69.9)  *41 (63.1)*  *59 (90.8)*  41 (44.1)  14 (15.1)  33 (35.5)  5 (5.4)  45 (48.4)  *32 (71.1)*  *1 (2.2)*  *25 (55.6)*  *2 (4.4)*  43 (46.2)  3 (3.2)  5 (5.4)  47 (50.0)  93 (100)  43 (46.2)  40 (43.0)  40 (43.0)  43 (46.2)  43 (46.2)  92 (98.9) | **0.01 (0.003-0.02)**  **0.08 (0.04-0.16)**  **5.40 (2.10-13.93)**  **0.18 (0.10-0.31)**  **0.19 (0.10-0.38)**  1.06 (0.66-1.39)  0.41 (0.16-1.06)  **0.04 (0.03-0.07)**  **0.22 (0.09-0.55)**  0.47 (0.06-3.68)  **3.65 (1.81-7.35)**  5.46 (0.83-35.83)  1.63 (0.95-2.81)  2.90 (0.56-15.05)  2.15 (0.70-6.60)  **3.74 (2.29-6.11)**  NA  **2.95 (1.39-6.23)**  **36.09 (18.82-69.20)**  **37.11 (19.25-71.52)**  **1.66 (1.07-2.57)**  **1.66 (1.07-2.57)**  0.48 (0.05-4.45) | **<0.001**  **<0.001**  **0.001**  **<0.001**  **<0.001**  0.821  0.066  **<0.001**  **0.001**  0.473  **<0.001**  0.077  0.077  0.204  0.184  **<0.001**  0.996  **0.005**  **<0.001**  **<0.001**  **0.007**  **0.007**  0.516 |
| Heart transplant  N admissions (n= 112)  Antibiotic use  BL AB (%)  *Narrow spectrum BL*^¥^  *Broad spectrum BL*^¥^  Penicillin AB (%)  Amoxicillin/clavulanic acid  Piperacillin/tazobactam  Flucloxacillin  Cephalosporin AB (%)  *First generation*  *Second generation*  *Third generation*  *Fourth generation*  Carbapenem AB (%)  Monobactam AB (%)  Tetracycline AB (%)  Quinolone AB (%)  Aminoglycoside AB (%)  Macrolide AB (%)  Lincosamide AB (%)  Clindamycin  Glycopeptides (%)  Vancomycin  Others (%) | 106 (94.6)  105 (99.1)  103 (98.1)  42 (40)  38 (35.8)  11 (10.4)  26 (24.5)  6 (5.7)  104 (98.1)  103 (99)  3 (2.9)  19 (18.3)  1 (1)  20 (18.9)  106 (100)  0 (0)  17 (16)  0 (0)  9 (8.5)  2 (1.9)  2 (1.9)  34 (32.1)  34 (32.1)  106 (100) | 6 (5.6)  4 (66.7)  3 (75)  4 (100)  2 (33.3)  0 (0)  2 (33.3)  0 (0)  4 (66.7)  3 (75)  0 (0)  3 (75)  0 (0)  0 (0)  6 (100)  0 (0)  1 (16.7)  0 (0)  0 (0)  3 (50)  3 (50)  2 (33.3)  2 (33.3)  6 (100) | 0.01 (0-0.38)  0.02 (0-0.77)  NA  0.98 (0-0)  NA  1.79 (0.29-10.94)  NA  0.03 (0-0.34)  0.02 (0-0.67)  NA  13.34 (1.27-140.51)  NA  NA  NA  NA  1.37 (0.14-13.72)  NA  NA  66.07 (5.84-747.96)  66.07 (5.84-747.96)  1.13 (0.19-6.54)  1.13 (0.19-6.54)  NA | 0.012  0.035  NA  0.980  NA  0.530  NA  0.005  0.028  NA  0.031  NA  NA  NA  NA  0.789  NA  NA  0.001  0.001  0.893  0.893  NA |
| Lung transplant  N admissions (n= 294)  Antibiotic use  BL AB (%)  *Narrow spectrum BL*^¥^  *Broad spectrum BL*^¥^  Penicillin AB (%)  Amoxicillin/clavulanic acid  Piperacillin/tazobactam  Flucloxacillin  Cephalosporin AB (%)  *First generation*  *Second generation*  *Third generation*  *Fourth generation*  Carbapenem AB (%)  Monobactam AB (%)  Tetracycline AB (%)  Quinolone AB (%)  Aminoglycoside AB (%)  Macrolide AB (%)  Lincosamide AB (%)  Clindamycin  Glycopeptides (%)  Vancomycin  Others (%) | 251 (85.4)  250 (99.6)  14 (5.6)  42 (16.8)  215 (85.7)  170 (67.7)  108 (43)  31 (12.4)  231 (92)  217 (93.9)  10 (4.3)  61 (26.4)  1 (0.4)  163 (64.9)  4 (1.6)  10 (4)  86 (34.3)  250 (99.6)  184 (73.3)  3 (1.2)  3 (1.2)  79 (31.5)  79 (31.5)  251 (100) | 43 (14.6)  43 (100)  14 (32.6)  1 (2.3)  28 (65.1)  10 (23.3)  24 (55.8)  3 (7)  35 (81.4)  24 (68.6)  1 (2.9)  20 (57.1)  2 (5.7)  34 (79.1)  3 (7)  4 (9.3)  22 (51.2)  43 (100)  41 (95.3)  9 (20.9)  9 (20.9)  14 (32.6)  14 (32.6)  43 (100) | NA  0.12 (0.05-0.32)  9.81 (1.29-74.78)  0.34 (0-0)  0.15 (0.07-0.32)  1.91 (0.98-3.75)  0.48 (0.14-1.66)  0.48 (0.19-1.23)  0.16 (0.06-0.45)  0.61 (0.07-5.09)  3.45 (1.58-7.51)  11.88 (0.96-146.57)  2.12 (0.94-4.78)  4.3 (0.65-28.6)  1.82 (0-0)  2.04 (1.04-3.98)  NA  8.43 (1.94-36.6)  20.12 (4.97-81.41)  20.12 (4.97-81.41)  0.92 (0.45-1.9)  0.92 (0.45-1.9)  NA | NA  <0.001  0.028  0.004  <0.001  0.058  0.244  0.126  0.001  0.645  0.002  0.054  0.071  0.132  0.375  0.037  NA  0.004  <0.001  <0.001  0.820  0.820  NA |
| Liver transplant  N admissions (n= 338)  Antibiotic use  BL AB (%)  *Narrow spectrum BL*^¥^  *Broad spectrum BL*^¥^  Penicillin AB (%)  Amoxicillin/clavulanic acid  Piperacillin/tazobactam  Flucloxacillin  Cephalosporin AB (%)  *First generation*  *Second generation*  *Third generation*  *Fourth generation*  Carbapenem AB (%)  Monobactam AB (%)  Tetracycline AB (%)  Quinolone AB (%)  Aminoglycoside AB (%)  Macrolide AB (%)  Lincosamide AB (%)  Clindamycin  Glycopeptides (%)  Vancomycin  Others (%) | 320 (94.7)  319 (99.7)  311 (97.4)  319 (100)  316 (98.8)  77 (24.1)  128 (40)  34 (10.6)  316 (98.8)  33 (10.4)  13 (4.1)  313 (99.1)  4 (1.3)  89 (27.8)  0 (0)  8 (2.5)  124 (38.8)  319 (99.7)  15 (4.7)  12 (3.8)  11 (3.4)  132 (41.3)  132 (41.3)  318 (99.4) | 18 (5.3)  9 (50)  4 (44.4)  8 (88.9)  7 (38.9)  3 (16.7)  6 (33.3)  0 (0)  5 (27.8)  3 (60)  0  4 (80)  0  6 (33.3)  0 (0)  2 (11.1)  17 (94.4)  18 (100)  2 (11.1)  3 (16.7)  3 (16.7)  18 (100)  18 (100)  18 (100) | 0 (0-0.02)  0.02 (0-0.09)  NA  0.01 (0-0)  0.63 (0.18-2.23)  0.76 (0.28-2.09)  NA  0 (0-0.02)  11.46 (1.74-75.47)  NA  0.07 (0-1.57)  NA  1.35 (0.49-3.76)  NA  6.44 (0-0)  26.79 (3.51-204.58)  NA  3.63 (0.71-18.52)  5.94 (1.44-24.48)  6.44 (1.56-26.57)  NA  NA  0.6 (0.07-4.99) | <0.001  <0.001  NA  <0.001  0.472  0.599  NA  <0.001  0.011  NA  0.094  NA  0.562  NA  0.032  0.002  NA  0.121  0.014  0.010  NA  NA  0.639 |
| Kidney transplant  N admissions (n= 416)  Antibiotic use  BL AB (%)  *Narrow spectrum BL*^¥^  *Broad spectrum BL*^¥^  Penicillin AB (%)  Amoxicillin/clavulanic acid  Piperacillin/tazobactam  Flucloxacillin  Cephalosporin AB (%)  *First generation*  *Second generation*  *Third generation*  *Fourth generation*  Carbapenem AB (%)  Monobactam AB (%)  Tetracycline AB (%)  Quinolone AB (%)  Aminoglycoside AB (%)  Macrolide AB (%)  Lincosamide AB (%)  Clindamycin  Glycopeptides (%)  Vancomycin  Others (%) | 386 (92.8)  382 (99)  366 (95.8)  137 (35.9)  180 (46.6)  40 (10.4)  89 (23.1)  38 (9.8)  364 (94.3)  343 (94.2)  16 (4.4)  49 (13.5)  1 (0.3)  39 (10.1)  1 (0.3)  2 (0.5)  28 (7.3)  189 (49)  18 (4.7)  18 (4.7)  18 (4.7)  112 (29)  112 (29)  382 (99) | 30 (7.2)  12 (40)  8 (66.7)  7 (58.3)  6 (20)  3 (10)  3 (10)  2 (6.7)  4 (13.3)  4 (100)  0 (0)  0 (0)  0 (0)  5 (16.7)  0 (0)  0 (0)  10 (33.3)  30 (100)  2 (6.7)  26 (86.7)  26 (86.7)  13 (43.3)  13 (43.3)  29 (96.7) | 0.01 (0.002-0.03)  0.1 (0.02-0.45)  2.09 (0.62-6.96)  0.25 (0-0)  0.87 (0.24-3.18)  0.34 (0.1-1.19)  0.65 (0.14-2.95)  0.01 (0.002-0.03)  NA  NA  NA  NA  1.47 (0.49-4.41)  NA  NA  6.91 (2.72-17.59)  NA  1.23 (0.25-6.12)  123.14 (37.44-405)  123.14 (37.44-405)  1.75 (0.8-3.85)  1.75 (0.8-3.85)  0.34 (0.03-3.49) | <0.001  0.003  0.232  0.005  0.839  0.092  0.579  <0.001  NA  NA  NA  NA  0.489  NA  NA  <0.001  NA  0.802  <0.001  <0.001  0.163  0.163  0.361 |
| Prophylactic indications^a^  N admissions (n= 7 423)  Antibiotic use  BL AB (%)  *Narrow spectrum BL*^¥^  *Broad spectrum BL*^¥^  Penicillin AB (%)  Amoxicillin/clavulanic acid  Piperacillin/tazobactam  Flucloxacillin  Cephalosporin AB (%)  *First generation*  *Second generation*  *Third generation*  *Fourth generation*  Carbapenem AB (%)  Monobactam AB (%)  Tetracycline AB (%)  Quinolone AB (%)  Aminoglycoside AB (%)  Macrolide AB (%)  Lincosamide AB (%)  Clindamycin  Glycopeptides (%)  Vancomycin  Others (%) | 6 912 (93.1)  6 842 (99.0)  *6 792 (99.3)*  *7 46 (10.9)*  1 217 (17.6)  772 (11.2)  557 (8.1)  94 (1.4)  6 601 (95.5)  *6 566 (99.4)*  *31 (0.5)*  *216 (3.3)*  *5 (0.1)*  147 (2.1)  0 (0.0)  19 (0.3)  526 (7.6)  167 (2.4)  64 (0.9)  163 (2.4)  163 (2.4)  254 (3.7)  254 (3.7)  1 523 (22.0) | 511 (7.88)  238 (50.2)  *226 (94.9)*  *39 (16.4)*  35 (6.8)  14 (2.7)  21 (4.1)  3 (0.6)  218 (42.7)  *213 (97.7)*  *3 (1.4)*  *15 (6.9)*  *0 (0.0)*  17 (3.3)  0 (0.0)  1 (0.2)  81 (15.9)  11 (2.2)  12 (2.3)  337 (65.9)  337 (65.9)  28 (5.5)  28 (5.5)  99 (19.4) | **0.008 (0.006-0.01)**  **0.12 (0.06-0.24)**  1.44 (0.97-2.14)  **0.37 (0.26-0.54)**  **0.25 (0.15-0.44)**  **0.53 (033-0.86)**  0.45 (0.14-1.42)  **0.02 (0.02-0.03)**  **0.21 (0.07-0.62)**  2.49 (0.74-8.41)  **1.85 (1.04-3.30)**  NA  **1.88 (1.08-3.26)**  NA  0.66 (0.09-4.96)  **2.39 (1.83-3.14)**  0.90 (0.49-1.67)  **2.55 (1.35-4.80)**  **91.02 (69.50-119.20)**  **91.02 (69.50-119.20)**  **1.71 (1.13-2.60)**  **1.71 (1.13-2.60)**  1.25 (0.85-1.84) | **<0.001**  **<0.001**  0.074  **<0.001**  **<0.001**  **0.010**  0.171  **<0.001**  **0.004**  0.141  **0.038**  0.995  **0.024**  NA  0.683  **<0.001**  0.75  **0.004**  **<0.001**  **<0.001**  **0.012**  **0.012**  0.253 |
| Prosthesis^b^  N admissions (n= 3 093)  Antibiotic use  BL AB (%)  *Narrow spectrum BL*^¥^  *Broad spectrum BL*^¥^  Penicillin AB (%)  Amoxicillin/clavulanic acid  Piperacillin/tazobactam  Flucloxacillin  Cephalosporin AB (%)  *First generation*  *Second generation*  *Third generation*  *Fourth generation*  Carbapenem AB (%)  Monobactam AB (%)  Tetracycline AB (%)  Quinolone AB (%)  Aminoglycoside AB (%)  Macrolide AB  Lincosamide AB (%)  Clindamycin  Glycopeptides (%)  Vancomycin  Others (%) | 2835 (91.7)  2 803 (98.9)  *2 792 (99.6)*  *90 (3.2)*  181 (6.4)  91 (3.2)  56 (2)  53 (1.9)  2 772 (97.9)  *2 770 (99.8)*  *4 (0.1)*  *28 (1.0)*  *1 (0.0)*  18 (0.6)  0 (0.0)  12 (0.4)  90 (3.2)  64 (2.3)  20 (0.7)  119 (4.2)  119 (4.2)  71 (2.5)  71 (2.5)  68 (2.4) | 258 (8.34)  89 (34.5)  *88 (98.9)*  *3 (3.4)*  6 (2.3)  5 (1.9)  1 (0.4)  1 (0.4)  85 (32.9)  *84 (98.8)*  *0 (0.0)*  *1 (1.2)*  *0 (0.0)*  1 (1.04)  0 (0.0)  1 (0.4)  22 (8.2)  6 (2.3)  5 (1.9)  200 (77.5)  200 (77.5)  8 (3.1)  8 (3.1)  4 (1.6) | **0.006 (0.004-0.009)**  0.32 (0.04-2.56)  1.05 (0.29-3.77)  **0.38 (0.16-0.87)**  0.63 (0.25-1.58)  0.25 (0.03-1.88)  0.23-0.03-1.65)  **0.01 (0.007-0.015)**  **0.05 (0.004-0.59)**  NA  1.74 (0.23-13.26)  NA  0.87 (0.11-6.83)  NA  0.83 (0.11-6.47)  **2.92 (1.77-4.81)**  1.24 (0.51-2.99)  3.56 (1.11-10.12)  **78.34 (55.10-111.11)**  **78.34 (55.10-111.11)**  1.66 (0.77-3.56)  1.66 (0.77-3.56)  0.63 (0.23-1.76) | **<0.001**  0.286  0.943  **0.023**  0.320  0.178  0.143  **<0.001**  **0.017**  0.997  0.593  0.974  0.893  NA  0.860  **<0.001**  0.633  **0.031**  **<0.001**  **<0.001**  0.195  0.195  0.379 |
| ICU admissions^a^  N admissions (n= 7 351)  Antibiotic use  BL AB (%)  *Narrow spectrum BL*^¥^  *Broad spectrum BL*^¥^  Penicillin AB (%)  Amoxicillin/clavulanic acid  Piperacillin/tazobactam  Flucloxacillin  Cephalosporin AB (%)  *First generation*  *Second generation*  *Third generation*  *Fourth generation*  Carbapenem AB (%)  Monobactam AB (%)  Tetracycline AB (%)  Quinolone AB (%)  Aminoglycoside AB (%)  Macrolide AB (%)  Lincosamide AB (%)  Clindamycin  Glycopeptides (%)  Vancomycin  Others (%) | 6901 (93.9)  6819(98.8)  5659(83)  4264(62.5)  4566(66.2)  2028(29.4)  2819(40.8)  402(5.8)  4785(69.3)  3422(71.5)  227(4.7)  1767(36.9)  92(1.9)  1823(26.4)  7(0.1)  95(1.4)  1607(23.3)  1650(23.9)  1280(18.5)  231(3.3)  230(3.3)  1840(26.7)  1712(24.8)  1986 (28.8) | 450 (6.1)  372(82.7)  226(60.8)  281(75.5)  191(42.4)  56(12.4)  148(32.9)  17(3.8)  257(57.1)  151(58.8)  21(8.2)  134(52.1)  12(4.7)  175(38.9)  6(1.3)  12(2.7)  209(46.4)  147(32.7)  106(23.6)  133(29.6)  133(29.6)  161(35.8)  151(33.6)  174 (38.7) | **0.05 (0.03-0.07)**  **0.26 (0.2-0.34)**  **1.95 (1.44-2.66)**  **0.21 (0.16-0.26)**  **0.24 (0.17-0.32)**  **0.61 (0.49-0.76)**  **0.59 (0.36-0.98)**  **0.42 (0.33-0.54)**  **0.38 (0.26-0.55)**  1.48 (0.91-2.41)  **2.32 (1.7-3.18)**  **2.09 (1.1-3.95)**  **1.77 (1.41-2.21)**  **10.65 (2.97-38.2)**  1.72 (0.91-3.25)  **2.64 (2.16-3.23)**  **1.4 (1.06-1.84)**  1.11 (0.85-1.45)  **13.01 (10.13-16.72)**  **13.08 (10.17-16.81)**  **1.61 (1.29-1.99)**  **1.57 (1.26-1.95)**  **1.57 (1.28-1.94)** | **<0.001**  **<0.001**  **<0.001**  **<0.001**  **<0.001**  **<0.001**  **0.041**  **<0.001**  **<0.001**  0.113  **<0.001**  **0.024**  **<0.001**  **<0.001**  0.094  **<0.001**  **0.016**  0.447  **<0.001**  **<0.001**  **<0.001**  **<0.001**  **<0.001** |
| AB, antibiotic; BL, beta-lactam; BLAL, beta-lactam allergy label; CI, confidence interval; ICU, intensive care unit; NA, not available (amount of subjects equals zero in at least one of the groups); OR, odds ratio; HR, hazard ratio; SD, standard deviation  ; **^§^**GEE used  ^a^ diagnoses, ^b^ type of prosthesis (hip or knee), and ^c^ type of transplant added to the model as confounders  ^¥^ **Narrow spectrum**: amoxicillin, amoxicillin/clavulanic acid, ampicillin, benyzlpenicillin, phenoxymethylpenicillin, benzathine benzyl penicillin, flucloxacillin, cefazolin, cefadroxil, cefuroxime; **Broad spectrum:** temocillin, piperacillin/tazobactam, cefoxitin, cefotaxime, ceftazidime with/without avibactam, ceftriaxone, cefepime, aztreonam, meropenem, imipenem/cilastatin | | | | |

**Table E7.** Associations of a reported **penicillin allergy label** with antibiotic use per study population with OR or β, as calculated via multiple regression (significant differences between those with and without a reported BLAL in **bold)**

| **Study population** | **No reported PenAL** | **Reported PenAL** | **OR or β (95% CI)** | ***p*-value** |
| --- | --- | --- | --- | --- |
| Total study population^a^ |  |  |  |  |
| N admissions (n= 23 842) | 22516 (94.4) | 1326 (5.6) |  |  |
| Antibiotic use |  |  |  |  |
| BL AB (%)**^§^** | 21218 (94.2) | 763 (57.5) | **0.07 (0.06-0.09)** | **<0.001** |
| *Narrow spectrum BL***^§^**^¥^ | *17337 (81.7)* | *407 (53.3)* | ***0.2 (0.16-0.24)*** | ***<0.001*** |
| *Broad spectrum BL***^§^**^¥^ | *9599 (45.2)* | *513 (67.2)* | ***3.01 (2.52-3.6)*** | ***<0.001*** |
| Penicillin AB (%)**^§^** | 13235 (58.8) | 300 (22.6) | **0.13 (0.11-0.16)** | **<0.001** |
| Amoxicillin/clavulanic acid**^§^** | 8099 (36) | 116 (8.7) | **0.14 (0.12-0.17)** | **<0.001** |
| Piperacillin/tazobactam**^§^** | 5776 (25.7) | 197 (14.9) | **0.48 (0.4-0.56)** | **<0.001** |
| Flucloxacillin**^§^** | 729 (3.2) | 17 (1.3) | **0.4 (0.25-0.66)** | **<0.001** |
| Cephalosporin AB (%)**^§^** | 12431 (55.2) | 472 (35.6) | **0.2 (0.16-0.26)** | **<0.001** |
| *First generation***^§^** | *9111 (73.3)* | *264 (55.9)* | ***0.39 (0.29-0.53)*** | ***<0.001*** |
| *Second generation***^§^** | *406 (3.3)* | *28 (5.9)* | *1.35 (0.89-2.05)* | *0.16* |
| *Third generation***^§^** | *3809 (30.6)* | *227 (48.1)* | ***2.09 (1.59-2.75)*** | ***<0.001*** |
| *Fourth generation* | *112 (0.9)* | *16 (3.4)* | ***2.72 (1.57-4.69)*** | ***<0.001*** |
| Carbapenem AB (%)**^§^** | 2911 (12.9) | 265 (20) | **1.91 (1.64-2.24)** | **<0.001** |
| Monobactam AB (%) | 13 (0.1) | 4 (0.3) | **4.60 (1.36-15.54)** | **0.014** |
| Tetracycline AB (%) | 167 (0.7) | 14 (1.1) | 1.36 (0.77-2.39) | 0.286 |
| Quinolone AB (%)**^§^** | 4821 (21.4) | 624 (47.1) | **3.81 (3.36-4.31)** | **<0.001** |
| Aminoglycoside AB (%) | 2937 (13) | 219 (16.5) | **1.37 (1.15-1.63)** | **<0.001** |
| Macrolide AB (%)**^§^** | 2625 (11.7) | 162 (12.2) | 1.03 (0.85-1.24) | 0.784 |
| Lincosamide AB (%)**^§^** | 682 (3) | 445 (33.6) | **16.66 (14.48-19.17)** | **<0.001** |
| Clindamycin**^§^** | 680 (3) | 445 (33.6) | **16.72 (14.53-19.24)** | **<0.001** |
| Glycopeptides (%)**^§^** | 2709 (12) | 197 (14.9) | **1.41 (1.19-1.66)** | **<0.001** |
| Vancomycin**^§^** | 2577 (11.4) | 190 (14.3) | **1.42 (1.2-1.68)** | **<0.001** |
| Others (%) | 4339 (19.3) | 307 (23.2) | **1.40 (1.22-1.60)** | **<0.001** |
| Therapeutic indications^a^ |  |  |  |  |
| N admissions (n= 16 641) | 15121 (90.9) | 1520 (9.1) |  |  |
| Antibiotic use |  |  |  |  |
| BL AB (%)**^§^** | 14560 (92.4) | 561 (64.1) | **0.08 (0.07-0.09)** | **<0.001** |
| *Narrow spectrum BL***^§^**^¥^ | *10730 (73.7)* | *213 (38)* | ***0.22 (0.18-0.26)*** | ***<0.001*** |
| *Broad spectrum BL***^§^**^¥^ | *8999 (61.8)* | *486 (86.6)* | ***3.06 (2.56-3.64)*** | ***<0.001*** |
| Penicillin AB (%)**^§^** | 12199 (77.4) | 276 (31.5) | **0.13 (0.11-0.16)** | **<0.001** |
| Amoxicillin/clavulanic acid**^§^** | 7438 (47.2) | 106 (12.1) | **0.14 (0.11-0.17)** | **<0.001** |
| Piperacillin/tazobactam**^§^** | 5346 (33.9) | 184 (21) | **0.48 (0.4-0.56)** | **<0.001** |
| Flucloxacillin**^§^** | 645 (4.1) | 15 (1.7) | **0.4 (0.24-0.66)** | **<0.001** |
| Cephalosporin AB (%)**^§^** | 6010 (38.1) | 286 (32.7) | **0.24 (0.19-0.3)** | **<0.001** |
| *First generation***^§^** | *2717 (45.2)* | *81 (28.3)* | ***0.49 (0.35-0.7)*** | ***<0.001*** |
| *Second generation***^§^** | *386 (6.4)* | *25 (8.7)* | *1.33 (0.86-2.07)* | *0.200* |
| *Third generation***^§^** | *3633 (60.4)* | *217 (75.9)* | ***2.24 (1.68-3.00)*** | ***<0.001*** |
| *Fourth generation***^§^** | *108 (1.8)* | *16 (5.6)* | ***2.67 (1.53-4.64)*** | ***0.001*** |
| Carbapenem AB (%)**^§^** | 2802 (17.8) | 253 (28.9) | **1.89 (1.62-2.21)** | **<0.001** |
| Monobactam AB (%) | 13 (0.1) | 4 (0.5) | **4.61 (1.36-15.57)** | **0.014** |
| Tetracycline AB (%) | 149 (0.9) | 13 (1.5) | 1.47 (0.82-2.65) | 0.198 |
| Quinolone AB (%)**^§^** | 4345 (27.6) | 555 (63.4) | **3.63 (3.22-4.1)** | **<0.001** |
| Aminoglycoside AB (%) | 2800 (17.8) | 209 (23.9) | **1.36 (1.15-1.62)** | **<0.001** |
| Macrolide AB**^§^** | 2572 (16.3) | 152 (17.4) | 1.02 (0.85-1.23) | 0.829 |
| Lincosamide AB (%) | 489 (3.1) | 146 (16.7) | **16.23 (14.12-18.66)** | **<0.001** |
| Clindamycin**^§^** | 487 (3.1) | 146 (16.7) | **16.29 (14.17-18.73)** | **<0.001** |
| Glycopeptides (%)**^§^** | 2501 (15.9) | 176 (20.1) | **1.4 (1.18-1.65)** | **<0.001** |
| Vancomycin**^§^** | 2369 (15) | 169 (19.3) | **1.41 (1.19-1.67)** | **<0.001** |
| Others (%)**^§^** | 3914 (24.8) | 284 (32.5) | **1.60 (1.38-1.85)** | **<0.001** |
| Prophylactic indications^a^ |  |  |  |  |
| N admissions (n= 7 423) | 7080 (95.4) | 343 (4.6) |  |  |
| Antibiotic use |  |  |  |  |
| BL AB (%)**^§^** | 6871 (98.7) | 209 (45.4) | **0.01 (0.01-0.01)** | **<0.001** |
| *Narrow spectrum BL***^§^**^¥^ | *6818 (99.2)* | *200 (95.7)* | ***0.15 (0.07-0.33)*** | ***<0.001*** |
| *Broad spectrum BL***^§^**^¥^ | *751 (10.9)* | *34 (16.3)* | *1.41 (0.95-2.1)* | *0.092* |
| Penicillin AB (%)**^§^** | 1221 (17.5) | 31 (6.7) | **0.37 (0.25-0.54)** | **<0.001** |
| Amoxicillin/clavulanic acid**^§^** | 774 (11.1) | 12 (2.6) | **0.24 (0.13-0.43)** | **<0.001** |
| Piperacillin/tazobactam**^§^** | 559 (8) | 19 (4.1) | **0.55 (0.34-0.88)** | **0.014** |
| Flucloxacillin | 94 (1.3) | 3 (0.7) | 0.47 (0.15-1.49) | 0.199 |
| Cephalosporin AB (%)**^§^** | 6626 (95.2) | 193 (42) | **0.02 (0.02-0.03)** | **<0.001** |
| *First generation***^§^** | *6590 (99.5)* | *189 (97.9)* | ***0.21 (0.07-0.63)*** | ***0.005*** |
| *Second generation* | *31 (0.5)* | *3 (1.6)* | *2.77 (0.82-9.33)* | *0.1* |
| *Third generation***^§^** | *219 (3.3)* | *12 (6.2)* | *1.62 (0.87-3.01)* | *0.127* |
| *Fourth generation* | *5 (0.1)* | *0 (0)* | *NA* | *0.996* |
| Carbapenem AB (%)**^§^** | 147 (2.1) | 17 (3.7) | **2.08 (1.22-3.53)** | **0.007** |
| Monobactam AB (%) | 0 (0) | 0 (0) | NA | NA |
| Tetracycline AB (%) | 19 (0.3) | 1 (0.2) | 0.72 (0.1-5.47) | 0.754 |
| Quinolone AB (%)**^§^** | 532 (7.6) | 75 (16.3) | **2.37 (1.8-3.14)** | **<0.001** |
| Aminoglycoside AB (%) | 168 (2.4) | 10 (2.2) | 0.91 (0.47-1.75) | 0.77 |
| Macrolide AB (%)**^§^** | 65 (0.9) | 11 (2.4) | **2.58 (1.33-4.98)** | **0.005** |
| Lincosamide AB (%)**^§^** | 196 (2.8) | 304 (66.1) | **75.76 (58.29-98.46)** | **<0.001** |
| Clindamycin**^§^** | 196 (2.8) | 304 (66.1) | **75.76 (58.29-98.46)** | **<0.001** |
| Glycopeptides (%)**^§^** | 257 (3.7) | 25 (5.4) | **1.56 (1.01-2.41)** | **0.043** |
| Vancomycin**^§^** | 257 (3.7) | 25 (5.4) | **1.56 (1.01-2.41)** | **0.043** |
| Others (%) | 1935 (22.0) | 87 (18.9) | 1.04 (0.79-1.40) | 0.774 |
| AB, antibiotic; BL, beta-lactam; BLAL, beta-lactam allergy label; CI, confidence interval; NA, not available (amount of subjects equals zero in at least one of the groups); OR, odds ratio; HR, hazard ratio; SD, standard deviation  §GEE used  a diagnoses added to the model as confounders  ¥ Narrow spectrum: amoxicillin, amoxicillin/clavulanic acid, ampicillin, benyzlpenicillin, phenoxymethylpenicillin, benzathine benzyl penicillin, flucloxacillin, cefazolin, cefadroxil, cefuroxime; Broad spectrum: temocillin, piperacillin/tazobactam, cefoxitin, cefotaxime, ceftazidime with/without avibactam, ceftriaxone, cefepime, aztreonam, meropenem, imipenem/cilastatin | | | | |

**Table E8.** Top ten most frequently used antibiotics in patients without *vs* with a beta-lactam allergy label (BLAL) for each condition separately. (Multiple prescriptions of the same antibiotic were counted only once per admission; non-betalactam antibiotics are displayed in **bold)**

a. Appendectomy

| **No BLAL (n= 1 789)** | **%** | **BLAL (n= 107)** | **%** |
| --- | --- | --- | --- |
| Cefazolin | 85.9 | **Metronidazole** | 78.5 |
| **Metronidazole** | 76.0 | **Clindamycin** | 60.7 |
| Amoxicillin/clavulanic acid | 23.5 | Cefazolin | 32.7 |
| Piperacillin/tazobactam | 11.5 | Levofloxacin | 25.2 |
| Levofloxacin | 8.11 | **Ornidazole** | 15.0 |
| **Ornidazole** | 2.91 | Amoxicillin/clavulanic acid | 7.48 |
| Meropenem | 2.63 | Meropenem | 7.48 |
| **Nifurtoinol** | 2.52 | Piperacillin/tazobactam | 7.48 |
| **Vancomycin** | 2.29 | **Vancomycin** | 4.67 |
| **Amikacin** | 1.84 | **Nitrofurantoin** | 3.74 |
| BLAL, beta-lactam allergy label |  | | |

b. Knee- or hip prosthesis

| **No BLAL (n = 2 835)** | **%** | | | **BLAL (n= 258)** | **%** |
| --- | --- | --- | --- | --- | --- |
| Cefazolin | | 97.7 | | **Clindamycin** | **77.5** |
| **Clindamycin** | | **4.19** | | Cefazolin | 32.6 |
| Amoxicillin/clavulanic acid | | 3.21 | | **Levofloxacin** | **6.20** |
| **Levofloxacin** | | **2.82** | | **Vancomycin** | **3.10** |
| **Vancomycin** | | **2. 50** | | **Amikacin** | **1.94** |
| Piperacillin/tazobactam | | 1.97 | | **Moxifloxacin** | **1.94** |
| Flucloxacillin | | 1.87 | | Amoxicillin/clavulanic acid | 1.94 |
| **Amikacin** | | **1.41** | | **Azitromycin** | **1.55** |
| **Gentamicin** | | **1.31** | | **Gentamicin** | **1.16** |
| **Sulfamethoxazole/trimethoprim** | | **0.95** | | **Nitrofurantoïne** | **0.78** |
| BLAL, beta-lactam allergy label | | |  | | |

c. Coronary artery bypass grafting

| **No BLAL (n= 2 288)** | **%** | | **BLAL (n= 146)** | **%** |
| --- | --- | --- | --- | --- |
| Cefazolin | 98.7 | | Cefazolin | 64.4 |
| Piperacillin/tazobactam | 12.9 | | **Clindamycin** | 49.3 |
| **Levofloxacin** | 11.6 | | **Levofloxacin** | 18.5 |
| Amoxicillin/clavulanic acid | 11.4 | | **Vancomycin** | 10.3 |
| **Vancomycin** | 6.21 | | Piperacillin/tazobactam | 8.22 |
| Ceftazidime | 4.28 | | Moxifloxacin | 5.48 |
| Meropenem | 3.58 | | Ceftazidime | 5.48 |
| Amoxicillin | 1.92 | | Meropenem | 5.48 |
| Ceftriaxone | 1.70 | | Ceftriaxone | 4.11 |
| Flucloxacillin | 1.44 | | Flucloxacillin | 1.37 |
| BLAL, beta-lactam allergy label | |  | | |

d. Pneumonia

| **No BLAL (n= 13 536)** | **%** | **BLAL (n= 897)** | **%** |
| --- | --- | --- | --- |
| Amoxicillin/clavulanic acid | 50.5 | **Moxifloxacin** | 46.5 |
| Piperacillin/tazobactam | 36.0 | Meropenem | 29.9 |
| Meropenem | 17.9 | Piperacillin/tazobactam | 22.4 |
| Cefazolin | 14.6 | **Levofloxacin** | 19.1 |
| **Vancomycin** | 14.4 | **Vancomycin** | 17.6 |
| Amoxicillin | 14.1 | Ceftriaxone | 16.5 |
| **Levofloxacin** | 13.6 | Amoxicillin/clavulanic acid | 14.3 |
| **Moxifloxacin** | 13.2 | **Clindamycin** | 13.7 |
| Ceftriaxone | 13.1 | **Amikacin** | 11.3 |
| **Clarithromycin** | 11.4 | **Sulfamethoxazole/trimethoprim** | 8.9 |
| BLAL, beta-lactam allergy label | | | |

e. Pyelonephritis

| **No BLAL (n= 1145)** | | **%** | **BLAL (n= 83)** | **%** |
| --- | --- | --- | --- | --- |
| Cefotaxime | | 39.7 | **Levofloxacin** | 67.5 |
| Levofloxacin | | 39.3 | **Amikacin** | 59.0 |
| **Amikacin** | | 33.0 | Ceftriaxone | 21.7 |
| Amoxicillin/clavulanic acid | | 28.2 | **Vancomycin** | 16.9 |
| Amoxicillin | | 18.2 | Meropenem | 14.5 |
| Piperacillin/tazobactam | | 14.3 | Temocillin | 14.5 |
| Temocillin | | 12.6 | Amoxicillin/clavulanic acid | 14.5 |
| Ceftriaxone | | 9.17 | **Clindamycin** | 13.3 |
| Meropenem | | 8.65 | Piperacillin/tazobactam | 13.3 |
| Cefazolin | | 8.56 | Cefotaxime | 9.64 |
| BLAL, beta-lactam allergy label |  | | | |

f. Transplant

| **No BLAL (n= 1028)** | **%** | | **BLAL (n= 93)** | **%** |
| --- | --- | --- | --- | --- |
| **Sulfamethoxazole/trimethoprim^§^** | | **96.2** | **Sulfamethoxazole/trimethoprim^§^** | **97.8** |
| Cefazolin | | 66.3 | **Tobramycin** | **95.7** |
| **Tobramycine** | | **66.1** | **Colistin** | **67.7** |
| **Colistin** | | **64.1** | **Vancomycin** | **46.2** |
| Amoxicillin | | 33.9 | Meropenem | 45.2 |
| **Vancomycin** | | **33.0** | **Azitromycin** | **44.1** |
| Piperacillin/tazobactam | | 32.8 | **Clindamycin** | **43.0** |
| Cefotaxime | | 31.8 | **Levofloxacin** | **43.0** |
| Ampicillin | | 28.4 | Piperacillin/tazobactam | 35.5 |
| Meropenem | | 28.3 | Cefazolin | 34.4 |
| BLAL, beta-lactam allergy label  § Prophylaxis for *Pneumocystis jirovecii* pneumonia (PJP) | | | | |

g. ICU admissions

(including AB administered at other departments during the same hospital admission)

| **No BLAL (n= 6 901)** | **%** | **BLAL (n= 450)** | **%** |
| --- | --- | --- | --- |
| Cefazolin | 49.6 | Meropenem | 38.4 |
| Piperacillin/tazobactam | 40.8 | Cefazolin | 33.6 |
| Amoxicillin/clavulanic acid | 29.4 | **Vancomycin** | 33.3 |
| Meropenem | 26.4 | Piperacillin/tazobactam | 32.7 |
| Amoxicillin | 25.6 | **Levofloxacin** | 31.6 |
| **Vancomycin** | 24.8 | **Clindamycin** | 29.6 |
| **Levofloxacin** | 18.8 | **Sulfamethoxazole/trimethoprim** | 20.2 |
| **Sulfamethoxazole/trimethoprim** | 15.5 | **Moxifloxacin** | 18.7 |
| **Colistin** | 12.3 | **Amikacin** | 18.0 |
| **Clarithromycin** | 11.6 | **Colistin** | 17.6 |
| BLAL, beta-lactam allergy label | | | |

h. Antibiotics administered at ICU only

| **No BLAL (n= 6 901)** | **%** | **BLAL (n= 450)** | **%** |
| --- | --- | --- | --- |
| Cefazolin | 42.1 | Meropenem | 31.8 |
| Piperacillin/tazobactam | 32.9 | Piperacillin/tazobactam | 26.9 |
| Meropenem | 23.0 | **Vancomycin** | 26.9 |
| **Vancomycin** | 19.3 | Cefazolin | 26.7 |
| Amoxicillin | 18.5 | **Clindamycin** | 18.2 |
| **Colistin** | 12.0 | **Colistin** | 17.1 |
| **Tobramycin** | 10.9 | **Tobramycin** | 14.7 |
| Amoxicillin/clavulanic acid | 10.4 | **Levofloxacin** | 11.3 |
| **Clarithromycin** | 10.0 | **Azithromycin** | 10.7 |
| **Sulfamethoxazole/trimethoprim** | 8.1 | Ceftriaxone | 10.0 |
| BLAL, beta-lactam allergy label | | | |

i. Therapeutic w/o transplant

| **No BLAL (n= 14 644)** | **%** | **BLAL (n= 976)** | **%** |
| --- | --- | --- | --- |
| Amoxicillin/clavulanic acid | 48.8 | **Moxifloxacin** | 43.1 |
| Piperacillin/tazobactam | 34.3 | Meropenem | 28.5 |
| Meropenem | 17.1 | **Levofloxacin** | 23.0 |
| **Levofloxacin** | 15.6 | Piperacillin/tazobactam | 21.5 |
| Amoxicillin | 14.4 | **Vancomycin** | 17.4 |
| Cefazolin | 14.0 | Ceftriaxone | 16.7 |
| **Vancomycin** | 13.9 | **Amikacin** | 15.2 |
| Ceftriaxone | 12.7 | Amoxicillin/clavulanic acid | 14.1 |
| **Moxifloxacin** | 12.2 | **Clindamycin** | 13.7 |
| **Amikacin** | 10.8 | Sulfamethoxazole/trimethoprim | 8.9 |
| BLAL, beta-lactam allergy label | | | |

**Table E9.** Number of antibiotics used per study population with IRR of poission regression (significant differences between the no BLAL and BLAL group in **bold)**

| **Study population** | **No reported BLAL** | **Reported BLAL** | **IRR (95% CI)** | **p-value** |
| --- | --- | --- | --- | --- |
| **Total^§^**^a^ | 2.42 (±1.91) | 2.61 (±2.12) | 0.98 (0.91-1.07) | 0.696 |
| **Therapeutic indications^§^**^a^ | 2.78 (±2.33) | 3.06 (±2.33) | 0.96 (0.87-1.06) | 0.425 |
| Pneumonia**^§^** | 2.57 (±1.88) | 2.77 (±2.14) | 1.01 (0.91-1.12) | 0.885 |
| Pyelonephritis**^§^** | 2.57 (±1.51) | 3.08 (±1.82) | 0.88 (0.62-1.25) | 0.478 |
| Transplantation**^b^** | 6.23 (±2.89) | 6.78 (±2.37) | 0.93 (0.74-1.16) | 0.497 |
| *Heart transplantation* | *5.5* (±2.22) | *5.67* (±1.51) | 1.17 (0.95-1.45) | 0.139 |
| *Lung transplantation* | *7.76* (±1.90) | *8.51* (±1.92) | 0.88 (0.66-1.18) | 0.393 |
| *Liver transplantation* | *8.38* (±2.36) | *6.89* (±2.56) | 0.78 (0.46-1.31) | 0.347 |
| *Kidney transplantation* | *3.97* (±2.26) | *4.77* (±1.31) | 1.42 (1.09-1.86) | **0.009** |
| **Prophylactic indications^§^**^a^ | 1.65 (±1.13) | 1.73 (±1.24) | 1.1 (1.02-1.19) | **0.019** |
| Appendectomy | 2.29 (±1.17) | 2.58 (±1.47) | 1.1 (0.92-1.31) | 0.291 |
| Coronary artery bypass grafting | 1.67 (±1.26) | 1.84 (±1.49) | 1.13 (1-1.27) | 0.055 |
| Prosthesis**^§^**^c^ | 1.24 (±0.74) | 1.32 (±0.67) | 1.07 (0.97-1.19) | 0.179 |
| *Hip prosthesis* | 1.27 (±0.80) | 1.40 (±0.77) | 0.95 (0.86-1.05) | 0.336 |
| *Knee prosthesis***^§^** | 1.21 (±0.68) | 1.26 (±0.57) | 1.15 (1.02-1.3) | **0.020** |
| **Patients with ICU admission(s)**^a^ | 3.58 (±2.60) | 4.15 (±2.84) | 0.91 (0.8-1.04) | 0.155 |

**^§^**GEE used; ^a^ diagnoses, ^b^ type of transplant, and ^c^ type of prosthesis (hip or knee) added to the model as confounders

**Table E10.** Duration of in-hospital antibiotic use per study population with IRR of poisson regression (significant differences between the no BLAL and BLAL group in **bold)**

| **Study population** | **No reported BLAL** | **Reported BLAL** | **IRR (95% CI)** | **p-value** |
| --- | --- | --- | --- | --- |
| **Total^§^**^a^ | 10.14 (±14.47) | 10.98 (±19.15) | 0.98 (0.94-1.04) | 0.564 |
| **Therapeutic indications^§^**^a^ | 13.09 (±) | 15.8 (±11) | 0.99 (0.93-1.04) | 0.641 |
| Pneumonia**^§^** | 12.88 (±15.24) | 13.82 (±19.74) | 1 (0.94-1.06) | 1.000 |
| Pyelonephritis**^§^** | 8.07 (±2.17) | 8.58 (±9.77) | 0.69 (0.52-0.9) | **0.006** |
| Transplantation^b^ | 24.26 (±25.35) | 32.92 (±41.81) | 1.1 (1.04-1.16) | **0.001** |
| *Heart transplantation* | *22.66* (±*38.16*) | *18.00* (±*8.37*) | 1.03 (0.89-1.19) | 0.715 |
| *Lung transplantation* | *31.38* (±*26.67*) | *47.72* (±*52.77*) | 1.16 (1.08-1.24) | **<0.001** |
| *Liver transplantation* | *27.21* (±*30.18*) | *29.44* (±*36.07*) | 1.01 (0.92-1.12) | 0.780 |
| *Kidney transplantation* | *19.59* (±*17.99*) | *16.93* (±*11.74*) | 1.03 (0.93-1.15) | 0.556 |
| **Prophylactic indications^§^**^a^ | 3.65 (±7.60) | 3.60 (±6.72) | 1.01 (0.9-1.12) | 0.916 |
| Appendectomy | 3.52 (±7.21) | 4.18 (±7.58) | 1.1 (0.95-1.28) | 0.217 |
| Coronary artery bypass grafting | 4.15 (±7.87) | 4.20 (±8.25) | 0.98 (0.79-1.22) | 0.861 |
| Prosthesis**^§^**^c^ | 3.32 (±7.61) | 3.02 (±3.91) | 0.95 (0.82-1.12) | 0.553 |
| *Hip prosthesis* | 3.23 (±7.60) | 3.19 (±4.94) | 1.05 (0.83-1.34) | 0.685 |
| *Knee prosthesis***^§^** | 3.40 (±7.61) | 2.89 (±2.77) | 0.88 (0.72-1.06) | 0.181 |
| **Patients with ICU admission(s)**^a^ | 16.24 (±21.03) | 21.04 (±31.50) | 1 (0.93-1.08) | 0.991 |

**^§^**GEE used; ^a^ diagnoses, ^b^ type of transplant, and ^c^ type of prosthesis (hip or knee) added to the model as confounders
